# Demographic interactions between the last hunter-gatherers and the first farmers

**DOI:** 10.1073/pnas.2416221122

**Published:** 2025-03-31

**Authors:** Alfredo Cortell-Nicolau, Javier Rivas, Enrico R. Crema, Stephen Shennan, Oreto García-Puchol, Jan Kolář, Robert Staniuk, Adrian Timpson

**Affiliations:** ^a^Department of Human Behavior, Ecology and Culture, Max Planck Institute for Evolutionary Anthropology, Leipzig 04103, Germany; ^b^Department of Archaeology, University of Cambridge, Cambridge CB2 3DZ, United Kingdom; ^c^Department of Economics, University of Bath, Bath BA2 7AX, United Kingdom; ^d^Institute of Archaeology, University College London, London WC1H 0PY, United Kingdom; ^e^Departament de Prehistòria, Arqueologia i Història Antiga, Universitat de València, València 46010, Spain; ^f^Institute of Archaeology of the Czech Academy of Sciences, Prague 3CR5+2P, Czech Republic

**Keywords:** demographic interaction, farming expansion, dynamic modelling, group competition, population dynamics

## Abstract

Demographic interaction processes play a pivotal role during episodes of cultural diffusion between different populations, particularly when these episodes can lead to competition for the same resources and geographic space. The diffusion of farming is one prototypical case within this broader scenario, where groups of incumbent hunter-gatherers occupied a space which would later be claimed by expanding farmers. In this work, we tackle such processes through a two-population mathematical model, where farmers and foragers compete and interact in the same geographic space. We present this work as a conceptual approach where, first, we assess the implications of our theoretical model and its general applicability and, second, we empirically test it on three case studies: Denmark, Eastern Iberia, and the island of Kyushu (Japan). While these regional case studies do not encompass the full range of processes observed in the interaction between migrant farmers and incumbent hunter-gatherers they provide reasonable variation to illustrate how our model can be fitted to a diverse range of empirical data and provide insights into these demographic processes. In particular, our theoretical model and case studies illustrate how endogenous interaction processes alone can explain the demographic fluctuations observed in the archaeological record during this transition, highlighting how these should be accounted for before invoking external forces as primary drivers.

## Significance

Demographic interactions play a pivotal role when two groups compete for the same space and resources, particularly when these interactions result in significant cultural change. We introduce a generalized mathematical model of demographic competition that can be used to explore a wide range of interactions between an incumbent and a migrant group. We demonstrate that when combined with simulation-based generative inference, our theoretical model can be fitted to empirical data. We illustrate the opportunities offered by this approach by investigating three archaeological case studies on the diffusion of farming, shedding light on the role played by population growth rates, cultural assimilation, and competition in shaping the demographic trajectories during the transition to agriculture.

The processes of cultural and technological transitions in past human groups have always been a core interest in anthropological and archaeological research. These transitions can happen either as a genuine internal development of such groups, through the adoption of cultural features from neighboring groups, or by population substitution or assimilation of expanding migrant populations. Here, we tackle these phenomena from the standpoint that demography plays a pivotal role when addressing interaction processes. We do so by deploying an adaptation of Lotka–Volterra (LV) models, a well-known mathematical description of interacting populations (see refs. 1–4). LV models are a common tool for studying the interactions of two or more groups competing for the same space or niche of resources. They are extensively used in ecology to model predator–prey interaction (see (5–8), but also in other fields, such as firms competing for market and innovation (9, 10), transnational economic convergence (11), or even green companies competing for fuel technologies (12). Archaeological applications of LV models are comparatively sparse. Early applications are closely aligned to the original formulation of the model, and depict interaction and demographic equilibriums of human populations with their prey (13, 14) or with their environment (15). Small modifications of this framing do also exist. For example, Spencer (16) modeled primary state formation by considering one population as “elite” and the other one as “commoner,” a path also supported by Flannery (17). While other applications of interaction models have appeared in the archaeological literature more recently (18–22), their use within the broader discipline is still comparatively rare, despite its potential to help elucidate demographic patterns and processes of interacting populations.

Here, we use LV models to analyze the demographic dynamics between early migrant communities of farmers and incumbent populations of hunter-gatherers during the transition to farming. These episodes have been studied mostly from the perspective of rates of expansion (e.g., refs. 23 and 24), by examining the role played by cultural differences (e.g., refs. 25 and 26) or by external drivers such as climate (e.g., refs. 27 and 28), assessing spatiotemporal patterns in the archaeological record (e.g., refs. 29–31) and, more recently, by examining aDNA data (e.g., refs. 32–35). Although these studies have been successful in expanding and consolidating our knowledge on the transition to farming, most of them focus either on exogenous elements that might have sparked or contributed to the change (36, 37) or on only one of the groups involved in the process (38, 39). This effectively neglects one of the key components to understand any potential interaction; that is, the relative sizes of the interacting groups. Furthermore, key demographic values governing these processes, such as growth rates, assimilation, and migration rates or mortality excess due to that same interaction are often overlooked. To account for this, we introduce a variant of the LV model. We first provide a theoretical exploration of the model, showing its details and grounds for generalization. Second, we apply it to three specific case studies (Iberia, Denmark, and the island of Kyushu, in Japan) where the expansion of farming was mainly due to incoming farmers, but where the exact details of this process are different enough to show the model’s performance in different empirical scenarios. We focus on purely endogenous demographic processes, while holding constant the effect of any external forces, such as climatic change and local suitability of different subsistence practices on demographic processes. We develop our interaction model from the basic premises of the LV model and explore its behavior space by iteratively holding constant key parameters, and see how the remaining parameters impact the demographic trajectories of the two populations. In their basic form, LV models consist of a pair of interrelated differential equations. Each of these represents the demographic dynamics of one population, including the effects derived from the interaction with the other population. Thus, because they reflect the reciprocal effects of each group, they become an ideal way to assess how both populations coevolve. Essentially, they rely on two key elements: intraspecific, or intragroup competition (the internal competition within one species or population), and interspecific, or intergroup competition (the competition between the two populations). The population dynamics generated by the interaction model lead to a coexistence of the two populations (in the long or short term) or the disappearance of one or both populations. In our case, all four possible outcomes are taken into account during the theoretical exploration. However, for the archaeological case studies, and in order to speed computation in the generative inference process (details in *SI Appendix*, S1), we consider only the steady state where the farmer population survives in the long term, in accordance with a very significant portion of the archaeological record. This is done for simplicity, and despite the acknowledgement that episodes of long-term coexistence or even of a reversal to foraging do exist.

Our model is tuned by six parameters: intrinsic growth population of the hunter-gatherers ($\gamma_{\mathrm{hg}}$), intergroup mortality of hunter-gatherers ($\delta_{\mathrm{hg}}$), intrinsic growth population of farmers ($\gamma_{f}$), intergroup mortality of farmers ($\delta_{f}$), external migration of the farmer population (μ) and an assimilation parameter (η), or the proportion of hunter-gatherers adopting farming. Furthermore, $\gamma_{f}$ and μ can be seen as hyperparameters defining ${\gamma \prime }_{f}$ (hence ${\gamma \prime }_{f}$ = $\gamma_{f}$ + μ), which accounts for the total growth of the farmers including endogenous growth and migration. It is worth noting that interspecific mortality refers to the mortality excess produced by the interaction with the opposing group. This does not specify the exact nature of such additional mortality, which could include direct conflict (40, 41), spread of disease [(42), but see also ref. 43], or increasing detrimental conditions of a specific ecological niche such as, for example, farmers eliminating potential hunting areas through fires, direct farming or otherwise (44, 45). Although we use the term “mortality” for convenience, we should note that the parameter effectively just captures the disappearance of individuals from the study region, which would also include processes such as emigration to other areas. Our model is also driven by two other factors, the initial population ratio between farmers and foragers, defined as $\rho =\frac{F_{t=1}}{{\mathrm{HG}}_{t=1}}$ where ρ is the ratio, F is the farming population, HG is the hunter-gatherer population and t is the timestep, and the carrying capacities of the two populations. For the sake of simplicity, we consider scenarios where the hunter-gatherers are at their maximum carrying capacity at the onset of the simulation and farmers are allowed to have increased carrying capacity as observed from the population curves of each group (*Materials and Methods*).

Our model construction prioritized the mathematical assessment of the impact of key growth parameters (namely $\delta_{\mathrm{hg}}$, $\delta_{f}$, and $\gamma_{\mathrm{hg}}$, $\gamma_{f}$), following the widely tested analytical framework of LV models. While the addition of both the assimilation (η) and migration (µ) parameters do not alter this mathematical framework, other potential parameterizations, such as changing carrying capacities, could alter the steady states (*SI Appendix*, S1). As for the values assigned to each parameter, we use ranges commonly suggested for late hunter-gatherers and early farmers in the archaeological literature (see details below and in *SI Appendix*, S1). We focused our model exploration and empirical testing on the scenarios that are most commonly observed in archaeological and ethnographic cases (i.e., coexistence of the two populations followed by a disappearance of hunter-gatherer groups). However, we note that our model can portray other dynamics, such as longer-term coexistence (e.g., refs. 46 and 47), including stable equilibrium, and “reversion” from hunter-gathering to farming (e.g., ref. 48). Here, we prioritized the detection of key features of only one possible scenario, which, nonetheless, can be characterized by a wide range of patterns (e.g., shorter or longer-term coexistence). We further acknowledge that the model can include additional factors, such as technological differences and a time-dependent change in resource availability. While these are potentially important aspects, we chose to focus on other aspects to reduce the complexity of the model, the computational cost in estimating parameters from empirical data (see below), and to introduce a model with a broader and more generalizable appeal. Therefore, and all in all, we consider the model in its current state as a compromise between its efficiency and its comprehensibility. There is of course room for further and specific hypothesis-based refinement from this solid ground.

The model produces time-series depicting changing population size of foragers and farmers for a given combination of parameters. We use Approximate Bayesian Computation (ABC) (49, 50) to estimate parameter combinations of our model generating time-frequency distributions of radiocarbon dates, with the closest fit to the observed archaeological record, as proxies for prehistoric population dynamics (51–53) (see *Materials and Methods* and *SI Appendix*, S1 for further details). We examined the robustness of our inferential framework by determining whether our approach can recover parameters from simulated data using comparable sample sizes as those of our case studies (*SI Appendix*, S2). Essentially, we present this work as a conceptual approach with three specific case studies, and advocate for its general value mainly through 1) the theoretical exploration proposed below and 2) the possibility to alter the ranges and priors of the parameters proposed, which would give place to very different outcomes without the necessity to alter the mathematical foundations of the model, nor the general analytical process presented here.

**Fig. 1.**
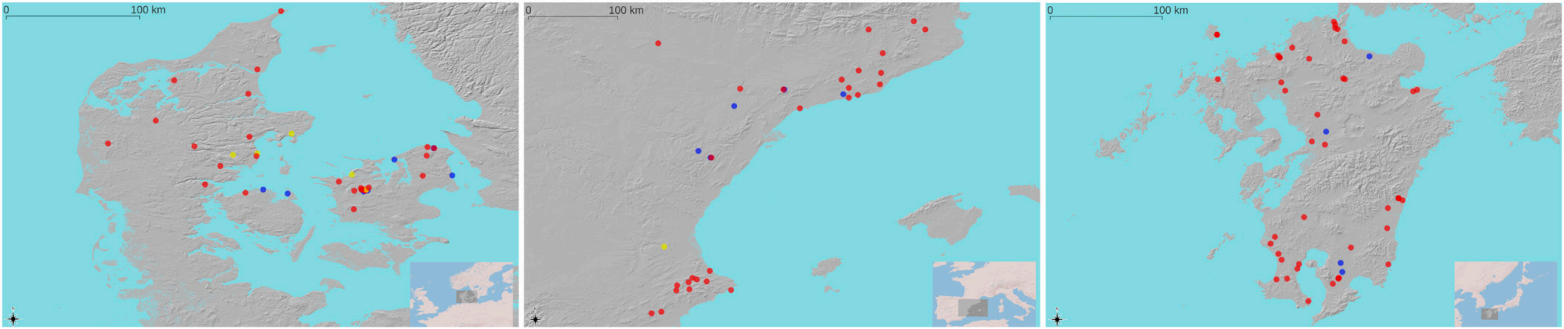
Map of the selected regions (Denmark, Iberia, and Kyushu). Red dots refer to sites associated with farming, blue dots are associated with hunter-gatherers, and yellow dots show dates for both of them.

We fit our model on datasets from three different archaeological contexts of farming transition: Eastern Iberia, southern Scandinavia, and the island of Kyushu (Japan) (Fig. 1). The priors and ranges for our parameters reflect the archaeological assumptions of the cases presented here, but they could be modified to include other putative dynamics. The three regions selected for this study differ in how the spread of farming was thought to be influenced by the incumbent hunter-gatherer population. For Mediterranean Iberia, the most prevalent hypothesis relies on the *Dual Model* (54–57), which considers two potential, not mutually exclusive, events occurring around 5600 BC: 1) an interaction between the last hunter-gatherers and the early farmers coupled with cultural assimilation of the former group, and 2) early farmers colonizing virtually empty lands with little to no admixture with local populations. In the Danish case, the southern farming frontier remained static for over a millennium from ~5200 BC before spreading rapidly as far north as central Sweden after ~4000 BC. There is extensive evidence of longer-term forager–farmer interaction in the intervening period, especially from ~4400 BC, but the aDNA evidence of recent years indicates that farming expansion from ~4000 BC was very rapid and was the result of demic diffusion (35, 58, 59). Finally, in the island of Kyushu, the prevalent hypothesis suggests multiple waves of demic diffusion from the Korean peninsula followed by interbreeding with the incumbent population of hunter-gatherers (60, 61) and a subsequent dispersal to the rest of the Japanese islands characterized by slow-downs, accelerations, and even episodes of a return to a hunting and gathering economy during the 1st millennium BC (62). Our results help us to understand how different aspects of demographic dynamics (e.g., population growth, migration, or intergroup violence) can explain the population patterns in these case studies while, through our theoretical exploration, we show how the model can be generalized to different contexts.

## Results

### Theoretical Model Exploration.

This section focuses on general values and wider applications of the model. The model proposed is complex, with six core parameters in addition to different initial settings of the two populations. We explore the behavior space of our model in two ways: (experiment 1) assessing a wide range of the population growth parameters where the intergroup mortality parameters are held constant at identical values for the two populations ($\delta_{\mathrm{hg}}=\delta_{\mathrm{hg}}=0.01$) and (experiment 2) exploring a range of the intergroup mortality parameters while holding the population growth parameters constant (${\gamma \prime }_{f}$ = 0.02 and $\gamma_{\mathrm{hg}}$ = 0.015). This leaves out two important settings: the assimilation parameter η and the initial population ρ. To also assess the impact of these parameters, we have fixed them at nine possible different scenarios, which broadly cover most of the realistic situations (Table 1). For each theoretical exploration we consider three temporal measures pertaining to specific key demographic events of interest: (event 1) the time taken until the farmers exceed the hunter-gatherers (or the first time-step where ρ>1); (event 2) the time taken until the farmers reach their carrying capacity and (event 3) the time taken until the hunter-gatherers disappear. In all cases, we have considered a maximum timespan of 1,500 y. We also show the analytically derived four equilibrium conditions of the model (*SI Appendix*, S1): 1) only hunter-gatherer population survives; 2) only farmer population survives; 3) stable coexistence of the two populations; and 4) only one population survives, conditional to the initial parameters of the model. Our model thus covers all possible equilibrium conditions, including both empirically observed and unobserved scenarios. Complementary images for the full exploration of the different scenarios can be found in *SI Appendix*, S3.

**Table 1.** Fixed values for *η* and initial population ratio to explore the parameters of population growth rate and interspecific mortality

Assimilation parameter (*η*)	*ρ* at *t* = 1
0	0.1
0	0.2
0	0.4
0.1	0.1
0.1	0.2
0.1	0.4
0.2	0.1
0.2	0.2
0.2	0.4

**Fig. 2.**
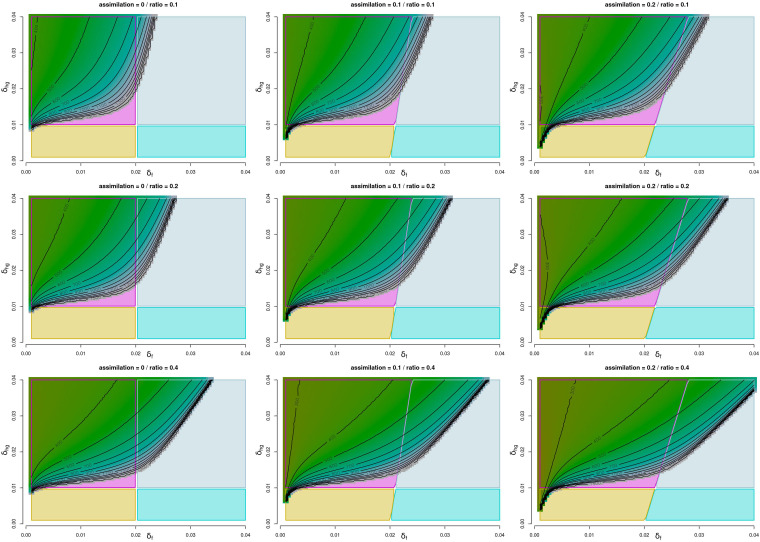
Experiment 1. Event 2. Time taken for the farmers to reach their carrying capacity under the full range of the interspecific mortality parameters. Growth rate parameter is set to 0.015 for the hunter-gatherers and 0.02 for the farmers. Assimilation parameter and initial population ratio follow the specifications of Table 1. Magenta indicates the parametric region where the farmers will be the only surviving population, yellow area indicates coexistence, in the blue area only hunter-gatherers will survive and in the gray area only one population survives, and which one does depends on the initial conditions. Isolines are set for every 100 y.

If we first focus on $\delta_{\mathrm{hg}}$ and $\delta_{f}$, with all things being equal, we can observe how higher intergroup mortality of both groups leads in general to a shorter time of diffusion of farming when both η and ρ are high (see also *SI Appendix*, S3). The time required for the farmers to reach their carrying capacity (Fig. 2), is also conditioned by interspecific mortality, with a shorter duration when $\delta_{\mathrm{hg}}$ is high and $\delta_{f}$ is low, all else being equal. This process is further accelerated with higher values of η and ρ. Indeed, farmers would reach their carrying capacity in broadly half of our considered timespan with maximum values of intergroup mortality for the two populations when η and ρ are high, while in the opposite scenario, this event might be delayed by several 100 y or not happen at all with much more frequency. This behavior is similar to the time taken by the farmers to overtake the hunter-gatherers population size. Although in this case, when both $\delta_{f}$ and $\delta_{\mathrm{hg}}$ are low, farmers exceed the hunter-gatherer population size without reaching their carrying capacity, while the latter group does not go extinct (*SI Appendix*, S3). Stable coexistence of the two groups is expected broadly when $\delta_{\mathrm{hg}}<0.01$ and $\delta_{f}<0.02$, whereas for $\delta_{\mathrm{hg}}>0.01$ and $\delta_{f}<\;0.02$ only the farming group would survive. However, we must note that, for higher values of η, both previous conditions increase their parametric space at $\delta_{\mathrm{hg}}$ at the cost of decreasing it at $\delta_{f}$ (in other words, they require an increased mortality of hunter-gatherers regarding farmers). This is the opposite situation where only the hunter-gatherers survive (broadly $\delta_{\mathrm{hg}}<\;0.01$ and $\delta_{f}>\;0.02$) and where both populations survive, but which one does depends on the initial conditions (broadly $\delta_{\mathrm{hg}}>\;0.01$ and $\delta_{f}>\;0.02$). As for the timing of the above-mentioned events, in general, when ρ = 0.1 (i.e., when the incumbent population of hunter-gatherers is 10 times larger than the migrant farming population), most parameter combinations lead to farmers overtaking the hunter-gatherer population size. This population overtake generally occurs mostly within 400 y from the start of the interaction, and hence before the farming population reaches its carrying capacity and before the hunter-gatherer population disappears. The time required by the farmers to reach their carrying capacity shows more variability within the parameter space explored here, but it rarely occurs before 400 y and usually, if the event has not occurred within 1,000 y of interaction, it would not happen at all within our time window of analysis. A similar pattern can be observed for the disappearance of the hunter-gatherers (*SI Appendix*, S3), which rarely occurs before 300 y (except when the initial proportion of farmers is high (i.e., ρ = 0.4), but also unlikely to happen at all within the window of analysis if it has not happened after 1,000 y of interaction.

**Fig. 3.**
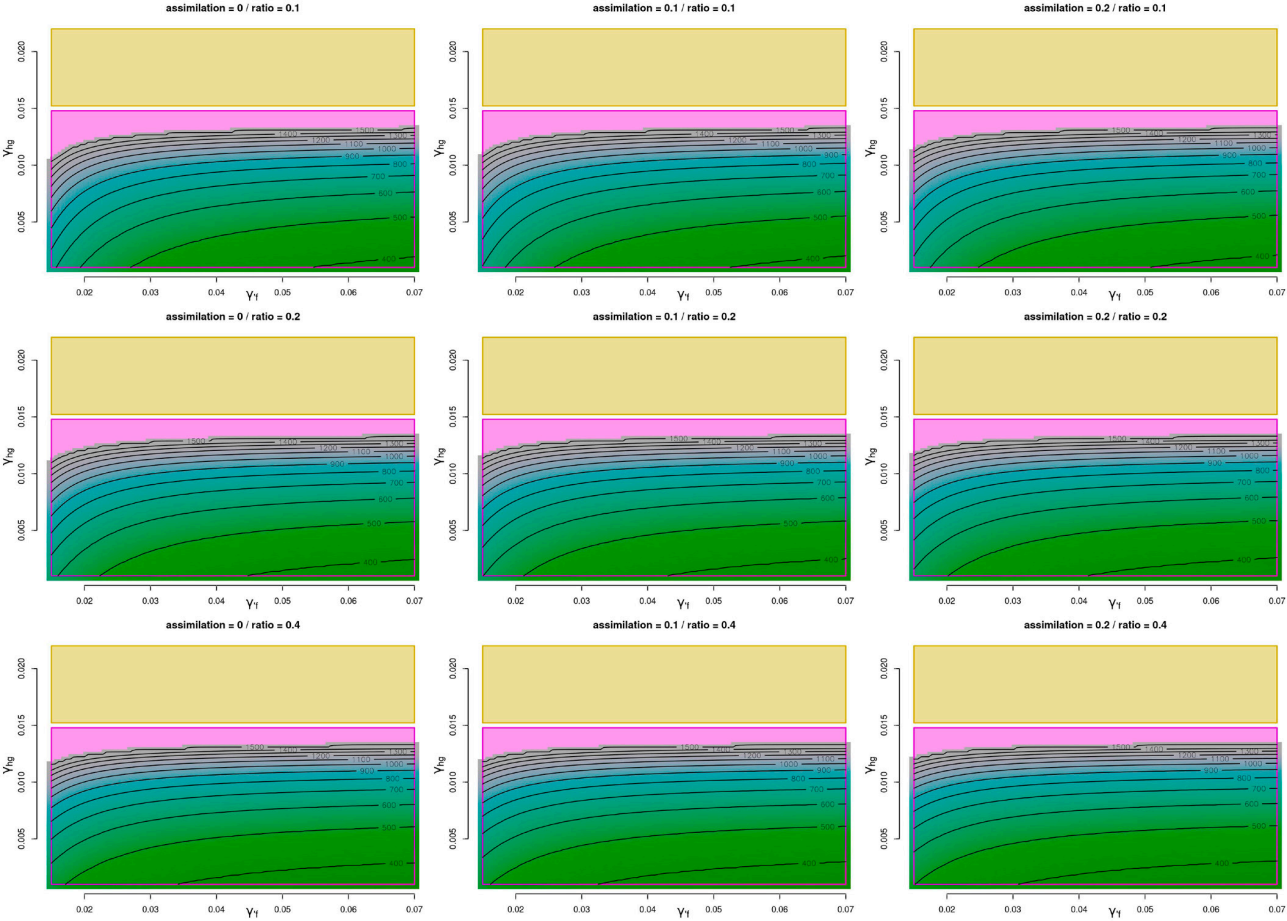
Experiment 2. Event 3. Time taken for the hunter-gatherers to completely disappear under the full range of the growth parameters. Interspecific mortality parameter is set to 0.01 for both populations. Assimilation parameter and initial population ratio follow the specifications of Table 1. Magenta indicates the parametric area where the farmers will be the only surviving population and yellow area indicates coexistence. Isolines are set for every 100 y.

As for experiment 2, here, $\delta_{\mathrm{hg}}$ and $\delta_{f}$ are both set to 0.01 and we explore $\gamma_{\mathrm{hg}}$ and ${\gamma \prime }_{f}$ (Fig. 3 and *SI Appendix*, S3), considering the time required to reach the same events as before. For this exploration, the range of the parameters is $\gamma_{\mathrm{hg}}$ = [0.001, 0.022] and ${\gamma \prime }_{f}$ = [0.015, 0.07], and is based on values suggested in the archaeological literature (e.g., refs. 63 and 64). Under these parameter settings, only two equilibriums are possible: either the farmer population is the only one surviving (when $\gamma_{\mathrm{hg}}<0.015$) or we have stable coexistence of both populations ($\gamma_{\mathrm{hg}}>0.015$). In terms of time to the events, farmers surpass the population size of hunter-gatherers under any possible range of parameters within the time range examined. In some extreme cases, when η and ρ are high, this can be as fast as within 50 or 40 y of interaction. Even when η and ρ are set to their lowest settings, this event still occurs within 600 y. When high values of ${\gamma \prime }_{f}$ are combined with low values of $\gamma_{\mathrm{hg}}$, the time for farmers to reach carrying capacity is around 300 to 400 y, and the time to hunter-gatherer extinction is about 400 to 500 y. In the first case, the event can be likely until some 800 y from the start of the interaction and in the second until circa 900 y.

While the above offers a wide range of situations applicable to most episodes of transition to farming, we have further explored some additional scenarios such as, for example, the case where hunter-gatherers would have higher carrying capacities than farmers. Since this is not an empirically common situation, and due to space constraints, this aspect is discussed in *SI Appendix*, S1. Briefly, however, in this case, with low $\gamma_{\mathrm{hg}}$ values, only farmers would survive for any nonzero populations, whereas for higher $\gamma_{\mathrm{hg}}$ values, there is the possibility of coexistence. All of this goes to show the versatility of the model and how it is applicable to a wide range of different contexts, where no single parameter can explain the final outcome, but rather it is the combination of parameters that can produce more or less unexpected results.

### Archaeological Case Studies.

**Fig. 4.**
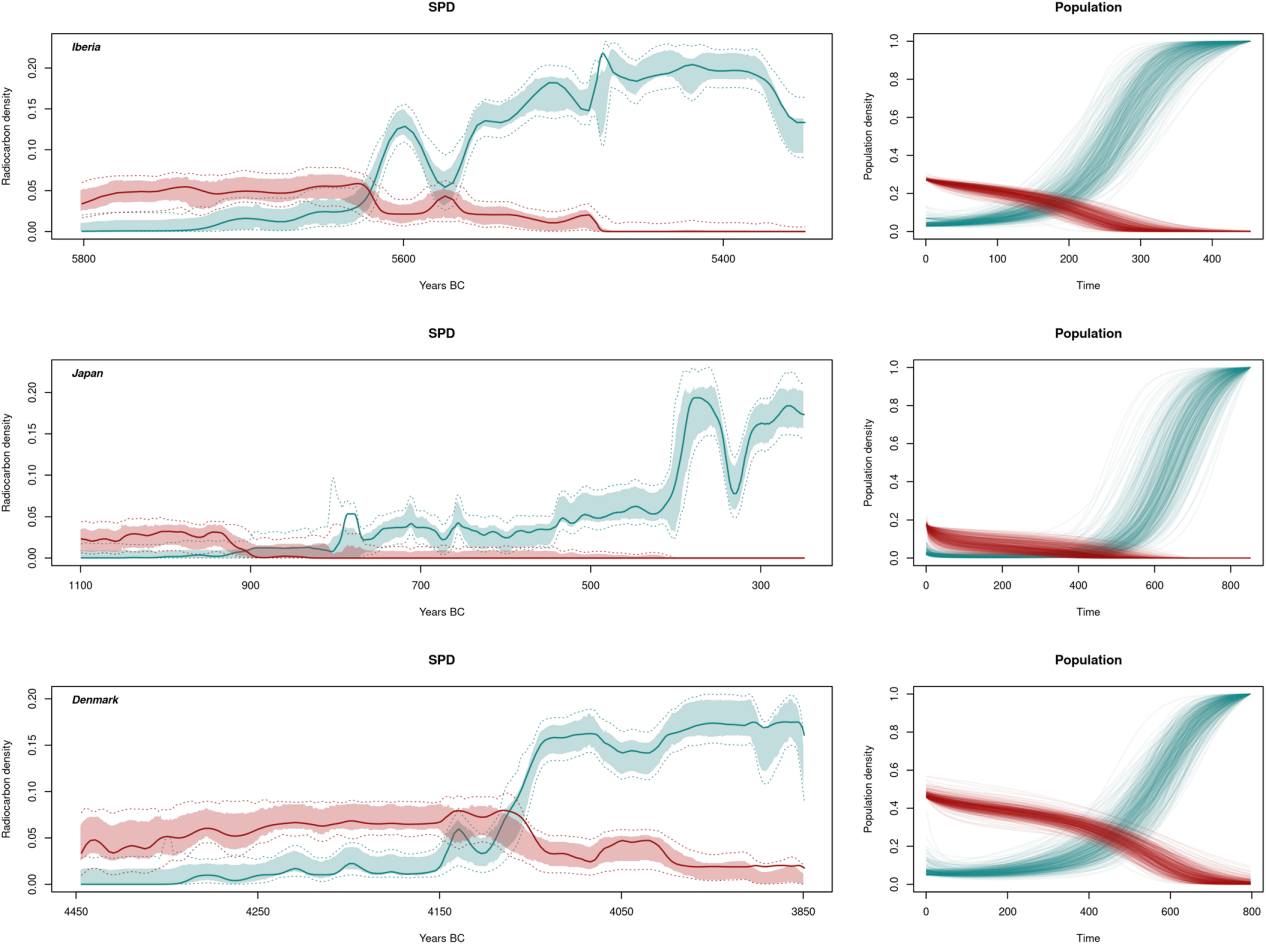
Population dynamics for the three areas considered. The left column shows the fitted SPDs produced by the model and the right column shows the actual population dynamics produced. On the fitted SPDs, lines represent the observed population and lighter bands represent the 95% HPDI. The right hand figures represent the accepted normalized population curves produced by the model. Red color represents the hunter-gatherers and green color the farmers.

The posterior predictive checks (Fig. 4) show that aside from minor deviations, the model can successfully describe the observed fluctuation over time in the density of radiocarbon dates associated with farming and foraging populations for all three case studies. It is interesting to note the differences in the initial population ratio between hunter-gatherers and farmers. In this regard, the initial values captured from our model return a median ρ of 0.14 [mean = 0.15 and 0.1 to 0.27 95% highest posterior density interval (HPDI)] for Denmark, 0.14 (mean = 0.17 and 0.1 to 0.37 95% HPDI) for Japan (consistent with values offered by ref. 60 and references therein) and 0.13 (mean = 0.14 and 0.1 to 0.26 95% HPDI) for Iberia. These values point to a situation where the detrimental consequences of the interaction on the hunter-gatherer population would start to be noticeable after ρ values of ~ 0.1 to 0.2 are reached.

**Fig. 5.**
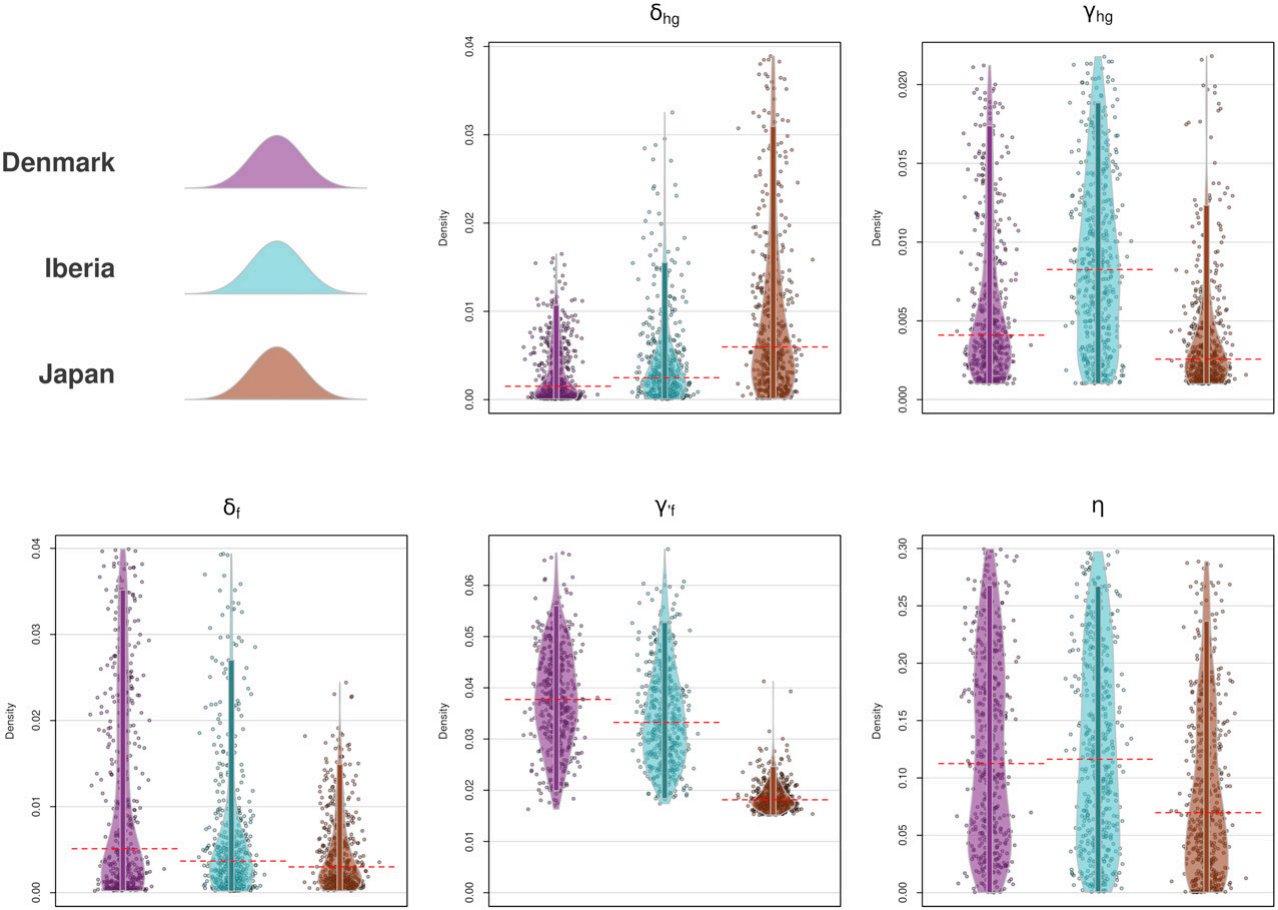
Posterior distribution of the parameters in the observed data per area. Bars indicate the 95% HPDI and dashed line represents the median. Y axes indicate the range of the uniform prior.

Considering the posterior distributions of the parameters (Fig. 5), most of them offer wide HPDIs. Acknowledging this, the intergroup mortality of the hunter-gatherers ($\delta_{\mathrm{hg}}$) shows a lower median in the European regions compared to Japan although there is overlap in their 95% HPDIs, this being compensated by opposite signatures in the population growth per area. It must be noticed that, for all areas, the median of the population growth rate for hunter-gatherers ($\gamma_{\mathrm{hg}}$) stays below 0.01, consistent with the well-known stall in the population growth of prehistoric hunter-gatherers (see refs. 58 and 59). Farmers’ interspecific mortality ($\delta_{f}$) is generally low, attending the available prior ranges, this more consistently in the Iberian and Japanese areas than in the Danish case, which shows a longer upper tail in its distribution. As for the farmers’ growth rate (${\gamma \prime }_{f}$), again the European areas show different signatures compared to Japan, with both regions showing higher values. In comparing the Danish and the Iberian cases, the latter suggests potentially a lower intergroup mortality. This can be related to a faster transition to agriculture, also coinciding with the shorter time-span for this same process in Iberia. Nevertheless, given that commonly observed values for early farmer growth rates typically do not exceed 0.035 (among others, 65), the migration of additional farmers from outside the geographical window of analyses has most likely played a key role in both Iberia and Denmark (33, 58, 59, 66). The Japanese case presents lower growth rates of the farming population compared to the other two regions examined, although within previous estimates (67) yielding an annual growth rate of 0.02 (0.018 to 0.028; 90% HPDI) after a shift in growth rate estimated around 715 cal BC (837 to 596 BC; 90% HPDI). Estimates of the assimilation rate also show lower median posterior values for Japan compared to the other two regions, albeit with considerably wide HPDI.

**Fig. 6.**
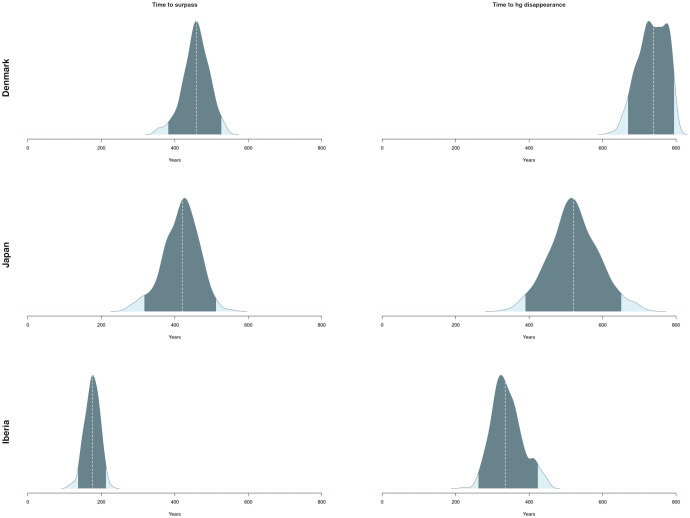
Time taken for different events reflecting the substitution of hunter-gatherers by farmers in each region. Darker areas indicate the 95% HPDI and dashed line represents the median.

The model reveals considerable variation in the duration of the process required for the replacement of hunter-gatherers by farmers (Fig. 6). This process is notably faster in Iberia with a mean time for the farmers to surpass the hunter-gatherer population of roughly 175 y (137 to 213; 95% HPDI) and a time needed for the hunter-gatherers to disappear of ca 341 y (262 to 424; 95% HPDI). For Denmark and Kyushu, while the time required for the farmers to exceed the hunter-gatherers yielded a similar posterior mean of 458 y (387 to 527; 95% HPDI) in the first case and 417 y for the second (315 to 513; 95% HPDI), hunter-gatherers in Denmark exhibit much more resilience than in the Japanese case taking on average roughly 200 more years to disappear (Denmark: posterior mean = 735 y, 95% HPDI: 669 to 795; Japan: posterior mean= 523 y 95% HPDI: 389 to 651). In this regard, it must be noted that Japanese hunter-gatherers disappear by the end of the window of analysis in every simulation and Iberian hunter-gatherers disappear in the vast majority of cases (~97.8%). However, the percentage of hunter-gatherers disappearing by the end of our time window in the Danish area is 44.6%. This means that, for more than half of the parameter values accepted, hunter-gatherers would still have a noticeable population after the time period considered for the study, thus envisaging a potential long-term coexistence.

## Discussion

### Empirical Case Studies.

Focusing on how this information translates into our current archaeological knowledge, the posterior estimates of the model parameters show a stronger resemblance between the Iberian and the Danish cases, albeit with some notable differences. In the Iberian case, our analyses showed how hunter-gatherer groups were present during the 7th millennium cal BC, continuing in the first half of the 6th millennium cal BC, with a drop contemporary with the arrival of farming around 5600 to 5500 cal BC. The main process of substitution or adoption of farming is relatively fast here, considered to span 400 y at maximum (68), a value consistent with our estimates. In contrast, the Danish case presents a significantly longer process of population interaction, estimated to be around 800 y in our model, with the transition period now estimated to start between 4085 and 3796 cal. BC (95.4%) and end between 3735 and 3458 cal. BC (95.4%), or a duration of up to ~600 y (35). Notwithstanding these details, the spread of farming into Scandinavia was most likely the result of a demic expansion during the late 5th millennium (69). It is worth highlighting some of the differences observed in the posterior parameters of these two regions. While in both cases we observe low growth rates for the incumbent hunter-gatherers, the intergroup mortality (either actual mortality or migration from the area) of the Danish farmers is slightly higher than that estimated for Iberia. This can be put in relation with the longer time that the process took in the Scandinavian region, since it would translate into a diminished pressure against hunter-gatherers on the hypothetical frontier which, in turn, could lead to a deferred and longer process of farmer dispersal and hunter-gatherer incorporation. Coupled with the slightly higher values for farming growth rate in the Danish case, once farmers are less affected by forager interference (because the hunter-gatherer population has decreased), they can experience a rapid population increase as observed in the SPD and the archaeological literature (35). Thus, for the Danish area, our model could be pointing to a situation of highly dynamic farming communities, in terms of mobility, which could also include internal movement (70). This dynamism is less feasible in Iberia, due to the essentially maritime character of the Neolithization process here (see ref. 71). Although contact and transmission patterns between the Southern coast of France and the Iberian Eastern façade are generally accepted (see refs. 72–74 and references therein), the lack of an extensive land support would limit the consistency of internal migratory patterns and networks of the early farming groups. Therefore, incoming farmers here could only either be unsuccessful in their spread or rapidly take over the new land found. This leaves the door open to failed attempts at introducing agriculture in the area, but it is difficult to assert, at the current state of knowledge, whether some of the earliest findings in the Valencian coast (see refs. 75 and 76) were indeed failed attempts or had a continuous connection with the later cardial groups, as recent studies of their lithic industry seem to indicate (77, 78). If there were indeed previous failed attempts, it is very likely that these are not part of the current archaeological record, and have not been recovered yet.

The Japanese case is slightly different. Here, we are considering a process spanning some 800 y and starting at around 1000 cal BC (62). As in the Iberian case, the main migratory component here is maritime (61). However, there are some notable differences in the estimated parameter values with the two European scenarios. More specifically, the higher interspecific mortality of the hunter-gatherers is coupled with low levels of cultural assimilation as well as low growth rates for the early farmers. This would imply that the farmer population had an impact on the demographic trajectory of the hunter-gatherer groups despite its smaller growth rate. It is worth noting that the dispersal of rice agriculture toward the southern portion of the island of Kyushu showed a significant delay (cf., ref. 62), most likely conditioned by poorer soil conditions that were less suitable for irrigated rice farming. This slowdown in the dispersal led to a relatively low growth rate during the early stages of interaction between migrant and local groups, which partly explains the temporal offset between the introduction of farming and the increase in local population sizes noted in previous studies (67). Our work suggests that, at least initially, the driver of the population replacement was the declining trend of the incumbent forager population.

### Theoretical Model.

With some notable exceptions (18–22), the application of interaction models in archaeology has been limited up to now. Here, we consider the last hunter-gatherers and early farmers in different parts of the world as distinct groups (or species, in the model’s terminology) competing for space and resources, which has a reciprocal impact on their population dynamics. If the extent of the impact that late hunter-gatherers had in their ecosystems is still under debate (although some recent studies (79) suggest that it might be higher than previously thought), it seems quite clear that early farmers substantially modified the environment where they initially settled (80, 81). In doing so, they would have modified the landscapes known to incumbent hunter-gatherers, provoking a demographic response. Such a response is the result of intergroup competition. Similarly, the demographic response here encapsulates a wide range of potential processes, from changes in life-history strategies to increased mortality due to disease or warfare, to emigration outside the geographic window of analyses. Some of these processes have been inferred in different regions, and they include relocation to inner and mountain areas by the late hunter-gatherers (82–84), but also violent encounters (40, 41). Conceptually, another important aspect to bear in mind is the limited sample available from the crucial period of early interaction between farmers and foragers. This demands caution regarding the data and scale for which the model is implemented. Although several of these phenomena (including reversions, etc.) might be observed locally, archaeological data often do not have the required resolution for a formal empirical analysis of such phenomena (see ref. 85). In other words, although the model has the analytical power to assess most contexts where two populations interact demographically, archaeological data may not (see *SI Appendix*, S2 for further discussion).

### Parameterization.

A caveat to have in mind is our definition of the assimilation parameter η. Conceptually, the parameter simply converts a constant proportion of the hunter-gatherers at any time *t*, into farmers. Our model does not define the exact nature of this process, which might occur via intermarriage, cultural exchange, or even independent innovation of agricultural practices. Additionally, we have not considered the potential transition from farming to foraging, as has been suggested for several contexts globally (48, 86). This is because we are only focusing on the average process of interaction, which can include temporary episodes of reversion, but which in the long run usually results in the shift to agricultural practices. We considered it necessary to account for the potential conversion to farming by incumbent hunter-gatherers due to the importance of this process within the overarching framework of interaction. However, the complexity of the process and all the potential factors involved would probably require a dedicated treatment beyond the scope of this article (see refs. 68, 73, 82, 87, and 88, among others). It is worth also discussing briefly how the growth rate for the early farmers has been defined within the model. This parameter is a combination of the intrinsic growth rate and the potential migration of other farmers from areas outside the window of analysis. In practice, this is irrelevant to our objectives, since we are only interested in quantifying how the population growth of the early farmers (regardless of what determines the growth) may affect the internal demographic dynamics of the last hunter-gatherers, but we understand how further research into this parameter may open insights into the demic component during the expansion of farming.

While the transition to farming is a multifaceted process, our study has shown that demographic interaction and cultural assimilation alone can potentially explain observed population dynamics. This conclusion does not naturally dismiss the direct or indirect role of external factors dictating the observed demographic trajectories, but demonstrates how our generative inference has highlighted a sufficiently robust alternative explanation that is worth investigating further. Demographic dynamics are in themselves an essential, and often overlooked, component of any process regarding human group substitution. In this regard, while it is true that in the present study, we have focused on three case studies (albeit quite diverse within the context of the adoption of farming), the model could be successfully applied not only to the transition to farming, but also to any context where different human groups compete for the same resources and where their interacting demographic dynamics are relevant to understand such process. Archaeologically speaking, this could be the case, just to name a few, of the substitution of Neanderthals by anatomically modern humans (89–91), the expansion of the Blades and Trapezes Complex (BTC) (92, 93) or even migratory movements in the Late Neolithic or Early Bronze Age (33, 94, 95). In these cases, the theoretical components of the model would remain the same as long as two populations compete for the same space and/or resources, the empirical limit depending on the resolution of the available data. Our mathematical model can capture an extremely versatile range of interaction simply by considering different parameter values, as shown in the theoretical exploration and the empirical cases. As usual, interpretation on whether and how the model can portray the demographic interaction observed in a particular region does not depend on the model itself, but on the current knowledge of the context. Furthermore, through this work, we hope to finally open the door for archaeology to the framework of dynamic modeling. Apart from the aforementioned cases, this has not yet caught on within the archaeological community at the same level that other families of models have, and we believe this is a potentially very useful tool for archaeologists not only because of its inferential power, shown here, but also to relate the discipline to broader modeling dynamics. By adopting such theoretical and methodological frameworks as part of its standard toolkit archaeology could benefit from a useful, widely tested, tool, in use since the first quarter of the 20th century, and from which other disciplines have already benefited. Ultimately, in order to investigate prehistoric transitional periods, a basic understanding of the demographic dynamics for the groups involved is key since any external factor which may be conditioning such transitions is going to be affected by the previous demographic relations of these groups. This demands population dynamics to be put in the frontline of archaeological research.

## Materials and Methods

### Data.

We have selected three areas with roughly equal sizes: Denmark (ca. 50,000 Km^2^), the island of Kyushu, in Japan (ca. 51,000 Km^2^), and the Eastern coast of Iberia (ca. 55,000 Km^2^). For each area, we have sampled the radiocarbon dates available involving the interaction process, where the dates have been labeled as hunter-gatherer/farmer, mostly according to the original publications. Additionally, all dates with SD higher than 120 y have been removed. We start the interaction process 100 y before the earliest farming date, after an initial assessment of potentially different starts through simulation (*SI Appendix*, S1).

### Summed Probability Distribution of Radiocarbon Dates (SPD).

With these dates we have built two different SPDs for each region, one belonging to the hunter-gatherers and one belonging to the farmers. The dates have been calibrated using the curve IntCal20 (96), and then they have been randomly thinned using bins of 20 y. Dates on the limit of the time range have only been accepted if more that 50% of their probability mass falls within the chronological window considered. Our final sample size consists of 102 dates for Denmark, 58 for Japan, and 69 for Iberia.

SPDs produced for the generative inference were based on the same number of dates as the observed data after random thinning. Dates were sampled using a discretized approach on the demographic curves produced by the model as the underlying probability distribution. In other words, we sampled the same number of dates as the target dataset, and then these would be assigned to one or another population binomially according to the density of each population at that specific time. Finally, the dates are uncalibrated and calibrated back to emulate the information loss in the calibration process (see ref. 53).

### The Model.

We have adapted the standard Lotka–Volterra model to our setting by 1) including a general interaction value into the equation, 2) a migration component only for the farming population, and 3) additionally considering the effect that the prey (hunter-gatherers) can have on the predator (farmers). In this present work, and due to the complexity of the model, we do not address particular intraspecific parameters (apart from the required migration component for the farmers), and we consider all other potential effects subsumed within the overall growth rate.

### Parameters.

Our model is based on the following continuous parameters, accounting for different aspects, as defined in Table 2. These parameters remain fixed for each simulation:

**Table 2.** Parameters used in the model

Parameter	Meaning
$\gamma_{\mathrm{hg}}$	Hunter-gatherer net population growth rate per year
$\gamma_{f}$	Farmer net population growth rate per year
$\delta_{\mathrm{hg}}$	Rate of hunter-gatherers disappearing per year due to interspecific competition
$\delta_{f}$	Rate of farmers disappearing per year due to interspecific competition
η	Proportion of hunter-gatherers becoming farmers
μ	Farmer’s migration

Furthermore, we should also account for

HG = Hunter-gatherer population.

F = Farmer population.

$K_{\mathrm{hg}}$ = Hunter-gatherer carrying capacity.

$K_{f}$ = Farmer carrying capacity.

t = Time in discrete calendar years.

ρ = Initial population ratio between the farmers and the hunter-gatherers.

### Initial Values.

The initial values for the hunter-gatherer and farming population sizes have been obtained by randomly selecting an initial value for the hunter-gatherer population and also randomly sampling *ρ* from the prior range from which the initial population of farmers is established. Additionally, $K_{\mathrm{hg}}$ is defined as the initial value of HG, since we assume that HG is at its carrying capacity without interference of the early farmers, and $K_{f}$ is computed as an inverse ratio using the ratio between the highest value of the hunter-gatherer SPD and the highest value of the farmer SPD.

Finally, we defined the start point and endpoint of the time-window of analysis of each case study based on contextual data. For the start point, we selected 100 y before the earliest radiocarbon date associated with farming, while for the endpoint, we selected a point time where the observed SPD suggests the farming population reaching its carrying capacity (see also *SI Appendix*, S1). In the former case, our inclusion criteria provide a compromise between the probability that early farmers were already present in the area, albeit undetected archaeologically, and pushing dates too far back without actual archaeological evidence of their presence. As for the endpoint, our criteria were based on our modeling scope limited to the initial uptake of farming which does not account for subsequent episodes of population decline that are likely to be independent from the interaction process with the forager population (97).

#### Mathematical formulation.

We use the following model:

$$\frac{\mathrm{dHG}}{\mathrm{dt}}=\gamma_{\mathrm{hg}}\cdot HG\cdot \left(1-\frac{\mathrm{HG}}{K_{\mathrm{hg}}}\right)-\delta_{\mathrm{hg}}\cdot HG\cdot F,$$

$$\frac{\mathrm{dF}}{\mathrm{dt}}=\gamma \prime_{f}\cdot F\cdot \left(1-\frac{F}{K_{f}}\right)+\eta \cdot I_{\mathrm{hg}}-\delta_{f}\cdot HG\cdot F,$$

where in the first equation the term $\gamma_{\mathrm{hg}}\cdot HG\cdot \left(1-\frac{\mathrm{HG}}{K_{\mathrm{hg}}}\right)$ indicates the hunter-gatherer population increase and $\delta_{\mathrm{hg}}\cdot HG\cdot F$ computes the effect of the interspecific mortality for hunter-gatherers. In the second differential equation ${\gamma \prime }_{f}\cdot F\cdot \left(1-\frac{F}{K_{f}}\right)$ quantifies the farmer population increase (where ${\gamma \prime }_{f}=\gamma_{f}+\mu$), $\delta_{f}\cdot HG\cdot F$ computes the effect of the interspecific mortality for farmers and $\eta \cdot I_{\mathrm{hg}}$ (where $I_{\mathrm{hg}}$ is the first term in the first differential equation and thus $I_{\mathrm{hg}}=\delta_{\mathrm{hg}}\cdot HG\cdot F$) quantifies the number of hunter-gatherers becoming farmers.

#### Approximate bayesian computation–sequential monte-carlo (ABC-SMC).

We use Approximate Bayesian Computation using a sequential Monte-Carlo algorithm. Archaeological applications of the method have already been explained elsewhere (e.g., refs. 66 and 98–100). In our case, as tolerance measure ϵ, we use the sum of the Euclidean distances between the simulated and observed SPDs of the hunter-gatherers and the farmers. To start the ABC-SMC process we initially develop a rejection algorithm on 15,000 simulations, where we select the 500 particles with the lowest ϵ to pass as the initial set of candidate values for the sequential Monte-Carlo process. This SMC process has been designed in six stages as follows: 1) each stage receives 500 candidate particles from the previous stage and iterates until it produces 500 new accepted particles with Euclidean distances lower than ϵ; 2) ϵ is updated at the beginning of each stage and is set at the first quantile of the Euclidean distances of the 500 candidate particles, and 3) in order to propose each set of parameter values from the candidate particle we use a uniform perturbation kernel $U\left[x-20\%,x+20\%\right]$ for each parameter (see ref. 101). In order to avoid local optima, we select the best 500 particles from the total generated with 100 randomized starts.

Based on archaeological and ethnographic literature, we have considered the following uniform priors (see references in *SI Appendix*, S1):

$$\gamma_{\mathrm{hg}}∼U\left[0.001,0.022\right],$$

$$\gamma_{f}∼U\left[0.015,0.035\right],$$

$$\delta_{\mathrm{hg}}∼U\left[0,0.04\right],$$

$$\delta_{f}∼U\left[0,0.04\right],$$

$$\eta ∼U\left[0,0.3\right],$$

$$\mu ∼U\left[0,0.035\right].$$

#### Tactical simulation.

We have performed a tactical simulation (*SI Appendix*, S2), repeating all the processes mentioned above with simulated data in order to assess the robustness of our inferential model. In other words, we assessed whether we were able to recover the parameters values we employed to generate our artificial datasets. We used this to assess the impact of different 1) sample sizes (in this case n = where 10, 25, 50, 100, 200, and 400 dates); 2) the time-window of analysis, using total interaction periods of 600, 800, and 1,000 y as well as potential starting points of 100, 200, 300, and 400 y before the earliest date for farmers; 3) total length of the process assessed, where we are constrained by the shape of our observed SPDs and we have decided to set the starting of the model 100 y before the earliest farming date for all areas; and 4) the precision and accuracy of the algorithm, when using different randomized starts, as mentioned above, has proven to return the best results.

The analysis has been produced using R statistical software 4.2.0 (102). We have used the R packages rcarbon (103), stats (102), foreach and doParallel (104), deSolve (105), and coda (106) for the analysis and colorRamps (107) for the figures.

## Supplementary Material

Appendix 01 (PDF)

## Data Availability

Code and .csv files data have been deposited in GitHub (https://github.com/acortell3/Demographic_interactions) (108) and Zenodo (https://doi.org/10.5281/zenodo.14992923) (109).
