# Supplementary material for: Demographic interactions between the last hunter-gatherers and the first farmers
Source: Proc Natl Acad Sci U S A. 2025 Mar 31;122(14):e2416221122. doi: 10.1073/pnas.2416221122 (PMC12002272; doi:10.1073/pnas.2416221122)
Supplement: Supplementary file 1 — Appendix 01 (PDF) [file pnas.2416221122.sapp.pdf]

SUPPLEMENTARY MATERIAL 1 (S1)

Methods

Data. Our initial intention was to focus on microregions where we can find a gap in the radiocarbon record between the last hunter-gatherers and early farmers, as is common in the Northern Mediterranean (1–3). However, this has not been possible due to the lack of radiocarbon data at a microregional level. Thus, we have decided to select larger regions but still following the main rationale that these regions should be small enough so that the human groups living in such areas could all be affected in some way by the interaction process. In this context, we have selected the areas of Denmark (~ 50000 Km^2), the island of Kyushu, in Japan (51000 Km^2) and the Eastern coast of Iberia (~ 55000 Km^2). For each area we have sampled the radiocarbon dates available that relate to the interaction process, where the dates have been tagged as hunter-gatherer/farmer, mostly according to the original publications. For this purpose, the data has been curated, reviewed and cleaned (with unreliable dates discarded) by experts in the region under study (SS for Denmark, ERC for Japan and OGP and ACN for Iberia). Additionally, all dates with standard deviation higher than 120 years have been removed. In order to tune the start and end of the process on the real SPDs we have used a simulated dataset (explained below). We have chosen as the most recent date the moment where the farming population reaches its peak, and we have set the start of the interaction process 100 years before the earliest farming date. The rationale for this is that we assume that we are missing the earliest dates of the farmers and the latest dates of the hunter-gatherers (since they are unlikely to be recovered by archaeologists due to their low frequency), but the process of interaction would already be underway, and is detectable by our model. Please note that we do not consider the bust in farming population after the initial peak (4), since our model is not prepared, nor designed, to account for busts of one single population without interaction.

SPDs. We have used these dates to build two different SPDs for each region, one belonging to the hunter-gatherers and one belonging to the farmers, where these SPDs would be used for the fitting of our model. To build these SPDs we have calibrated the dates using the curve Intcal 20 (5), and then these have been randomly thinned using bins of 20 years to avoid problems of overrepresentation of single sites (see (6)). Additionally, dates on the limit of the time range have only been accepted if more that 50% of their probability mass falls within the chronological window considered. After these processes, we have remaining a total of 102 dates for Denmark, 58 for Japan and 69 for Iberia. According to our previous simulations, explained below, and given our chronological span, there is no substantial difference in terms of fitting from 50 to 100 dates, so we consider the sample size appropriate.
As for the SPDs produced by the model. We generate a number of dates equal to the number of dates present in each specific dataset after random thinning, using a discretised approach on the curves produced by the model as the underlying probability distribution. In other words, we would sample the same number of dates as the target dataset, which would approach the population density resulting from the two populations of the model given the proposed parameters, and then these would be assigned to one or another population binomially according to the density of each population at that specific time. After we have the final number of dates, and their specific affiliations, these are uncalibrated and calibrated back to reproduce the radiocarbon error (6).

The model. Our model proposal is based on the Lotka-Volterra family of models (LV), also known as predator-prey models. These were developed originally and independently by Alfred J. Lotka and Vito Volterra in 1925 (7) and 1926 (8) respectively, and have been extensively used within the ecological literature until the present day. In a nutshell, this type of model considers the interactions of two populations, and measures the effect of one population on the other and vice versa.
The Lotka–Volterra predator–prey model consists of two differential equations, each describing the evolution of a population given a set of parameters and the interaction between populations.

The model predicts several long-term outcomes depending on the parameter values, from both populations co-existing to only one of them surviving in the long run.
We have adapted the standard LV model to our setting by (1) including a general interaction value in the equation, (2) a migration component only for the farming population and (3) considering the effect that the prey (hunter-gatherers) can have on the predator (farmers). As a final note before the technical details, these models usually rely on intraspecific values and interspecific values, where the first refers to the components determining the independent effect of different variables for one single population and the latter refers to the effects that the populations have on each other. In this present work, and due to the complexity of the model, we do not address particular intraspecific parameters (apart from the required migration component for the farmers), and we consider all other potential effects subsumed within the overall growth rate. That is, we focus on interspecific parameters, since our aim is to determine the effects of the interaction and not of specific elements affecting each population individually.

Parameters. Our model is based on the following parameters, accounting for different aspects:

γ_{hg} = Hunter-gatherer net population growth rate per year. This includes the intraspecific effects. This is a continuous parameter and $\gamma_{hg} > 0$.
γ_f = Farmer net population growth rate per year. This includes the intraspecific effects. This is a continuous parameter and $\gamma_f > 0$.
δ_{hg} = Rate of hunter-gatherer population disappearing per year due to interspecific competition. This is a continuous parameter and $\delta_{hg} > 0$.
δ_f = Rate of farmer population disappearing per year due to interspecific competition. This is a continuous parameter and $\delta_f > 0$.
η = This parameter has been used by Fenton et al. (9) as the amount of prey biomass assimilated per predator. In our case, we interpret it as the number of hunter-gatherers becoming farmers. We have not considered, at this point, the possibility of farmers becoming hunter-gatherers, since we believe that the overall net balance would probably favour the conversion of hunter-gatherers to farming. However, we acknowledge that this is potentially a factor for further study. This is a continuous parameter with theoretical range $[0,1]$.
μ = Migration component. This is a continuous parameter and,theoretically, $\mu > 0$, but in practice it is determined by the existing population at the origin areas. However, it has been treated as a general incoming value, and the population at the points of origin has not been considered at this stage.

Furthermore, we should also account for
HG = Hunter-gatherer population.
F = Farmer population.
K_{hg}= Hunter-gatherer carrying capacity.
K_f = Farmer carrying capacity.
t = Time in discrete calendar years. Time has been discretised for simulation purposes, where each year is one unit of time, and the output of the model is treated accordingly.

Regarding the initial population and carrying capacities, it is almost impossible and utterly speculative to infer an actual value for the initial population during the interaction process. Therefore, we have decided to randomise the values for the initial moment. To do this, we randomly select an initial value for the hunter-gatherer population and also randomly establish a ratio for hunter-gatherers/farmers, from which the initial population of farmers is established.

Additionally, K_{hg} is defined as the initial value of HG, since we assume that HG is at its peak without interference of the early farmers, and K_f is computed as an inverse ratio using the ratio between the highest value of the hunter-gatherer SPD and the highest value of the farmer SPD.

Model. We compute the hunter-gathererMesolithic population increase per year as the product of the net growth fate, current population and the logistic population growth equation

$$HG=\gamma_{hg}\cdot HG\cdot\left(1-\frac{HG}{K_{hg}}\right). \qquad [1]$$

We then subtract the effect of interaction with

$$I_{hg}=\delta_{hg}\cdot HG\cdot F\ . \qquad [2]$$

Since the change in Mesolithic population is given by $\frac{dHG}{dt}=HG-I_{hg}$, we have that the first differential equation is

$$\frac{dHG}{dt}=\gamma_{hg}\cdot HG\cdot\left(1-\frac{HG}{K_{hg}}\right)-\delta_{hg}\cdot HG\cdot F. \qquad [3]$$

The farmer population increase is computed in similar terms. First, we have the growth term

$$A=(\gamma|f+\mu)\cdot F\cdot\left(1-\frac{F}{K_f}\right), \qquad [4]$$

or

$$A=\gamma'_f\cdot F\cdot\left(1-\frac{F}{K_f}\right).$$

Notice that the farmer population growth includes the migration component. Therefore, the parameter accounting for the full farmer population growth is $\gamma'_f=\gamma_f+\mu$.
The subtraction effect is also similarly derived:

$$I_f=\delta_f\cdot HG\cdot F. \qquad [5]$$

However, in this case we add the effect of hunter-gatherer population becoming farmers by

$$E=\eta\cdot I_{hg}. \qquad [6]$$

Since the change in farmer population is given by, $\frac{dF}{dt}=A+E-I_f$, the second differential equation is

$$\frac{dF}{dt}=\gamma'_f\cdot F\cdot\left(1-\frac{F}{K_f}\right)+\eta\cdot I_{hg}-\delta_{hg}\cdot F\cdot HG. \qquad [7]$$

Combining both differential equations yields the following system of equations:

$$\begin{aligned}\frac{dHG}{dt}&=\gamma_{hg}\cdot HG\cdot\left(1-\frac{HG}{K_{hg}}\right)-\delta_{hg}\cdot HG\cdot F\\ \frac{dF}{dt}&=\gamma'_f\cdot F\cdot\left(1-\frac{F}{K_f}\right)+\eta\cdot I_{hg}-\delta_f\cdot HG\cdot F,\end{aligned} \qquad [8]$$

or simply

$$\begin{aligned}\frac{dHG}{dt}&=H-I_{hg}\\ \frac{dF}{dt}&=A+E-I_f.\end{aligned}$$

Equilibrium and analytical/numeric solution.
For Lotka-Volterra models such as ours, we can calculate what is the steady state of the system of equations, i.e. which populations survive after a large number of periods has elapsed.

From [8], we can rewrite the system of differential equations as

$$\begin{aligned}\frac{dHG}{dt}&=HG\left(\gamma_{hg}-\frac{\gamma_{hg}}{K_{hg}}HG-\delta_{hg}F\right)\\ \frac{dF}{dt}&=F\left(\gamma'_f-\frac{\gamma'_f}{K_f}F-\left(\delta_f-\eta\delta_{hg}\right)HG\right)\end{aligned} \qquad [9]$$

Notice both expressions are in form $\frac{dx}{dt}=x\left(a_x-b_x x-c_x y\right)$ for $x\in\{M,N\}$, $y\in\{M,N\}$. In this formulation, a_x represents the intrinsic growth rate of x, b_x its intrapopulation competition rate and c_x the interpopulation rate of x against y.

The system in [9] has four stable points written in the form (HG,F): (0,0), (K_{hg},0), (0,K_f) and

$$\left(\frac{\gamma_{hg}\frac{\gamma'_f}{K_f} - \gamma_{hg}\left(\delta_f - \eta\,\delta_{hg}\right)}{\frac{\gamma_{hg}\gamma'_f}{K_{hg}K_f} - \delta_{hg}\left(\delta_f - \eta\,\delta_{hg}\right)}, \frac{\gamma'_f\frac{\gamma_{hg}}{K_{hg}} - \gamma_f\,\delta_{hg}}{\frac{\gamma_{hg}\gamma'_f}{K_{hg}K_f} - \delta_{hg}\left(\delta_f - \eta\,\delta_{hg}\right)} \right)$$

. In (0,0) both populations die, in (K_{hg},0) and (K_f,0) only one population survives (HG and F respectively). Finally, in the fourth stable point both populations survive in the long run (co-existence).

Using the equations in [9] and known facts about LV models (see (10) for instance), we have the following result

Proposition 1. Define $\lambda_1 = \gamma_{hg} - \delta_{hg} K_f$ and $\lambda_2 = \gamma'_f - \left(\delta_f - \eta\,\delta_{hg}\right) K_{hg}$ and assume both initial starting populations are positive, then

1. If $\lambda_1 > 0$ and $\lambda_2 < 0$ then only population HG survives.
2. If $\lambda_1 < 0$ and $\lambda_2 > 0$ then only population F survives.
3. If $\lambda_1 > 0$ and $\lambda_2 > 0$ then there is co-existence.
4. If $\lambda_1 < 0$ and $\lambda_2 < 0$ then only one population survives, where which one does so depends on the initial population values.

We know based on archaeological knowledge that, for the cases studied, unequivocally and regardless of initial population levels, only the farming strategy survives in the long run and the hunter gatherers' one disappears. Thus, in the simulations we only consider parameter constellations such that $\lambda_1 < 0$ and $\lambda_2 > 0$. This allows us to greatly increase our speed and efficiency through bypassing the consuming process of calibrating radiocarbon curves that we know beforehand would not fit the data. However, if any researcher interested in reusing this model had reasons to believe that this condition is not met, and the substitution of hunter-gatherers by farmers is not the only possible option within the context of their study, this can easily be modified or removed by commenting (or adapting) the following lines in the provided code:

```
## Proposition 1 in sup mat: Correction for discarding unrealistic
## parameterisations


## Establish values of lambda1 and lambda2
Li <- possible_pars["Gm"]-(possible_pars["Dm"]*possible_pars["Kn"])
Lii <- (possible_pars["Gn"]+possible_pars["m"])-((possible_pars["Dn"]-
        (possible_pars["e"]*possible_pars["Dm"]))*possible_pars["Km"])

## Select parameterisation only if not unrealistic
if ((Li < 0 & Lii > 0) | (Li < 0 & Lii < 0)){
  Prior_sim_s_pars <- possible_pars
  state <- c(M = M, N = N)}
```

Even though the asymptotic (*i. e.* long run) properties of the system in [9] are well understood (see proposition), there is no known way to understand its dynamic evolution analytically. This is

because given the high complexity of the LV systems such as the one in [9] there is no known analytical solution for the trajectory of their equations. Nevertheless, we can use numerical calculations to compute such trajectories.

For example, if we set up parameters such that $\lambda_1 < 0$ and $\lambda_2 > 0$, we have a situation similar to the following in Supplementary Figure S1

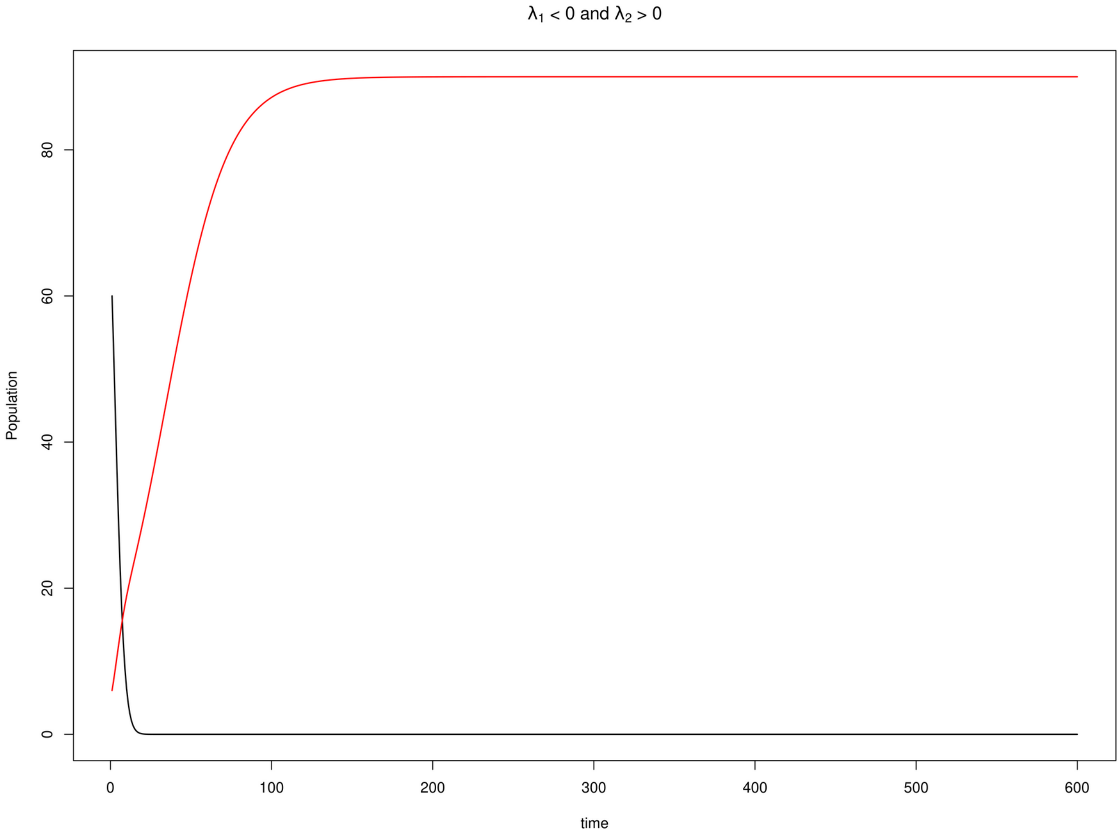

Supplementary Figure S1. With this parameters, the farmer population (red) exceeds the hunter-gatherer population (black).

Whereas considering parameters instead such that $\lambda_1 < 0$ and $\lambda_2 < 0$ with the same initial conditions the evolution could look like the following supplementary figure S2

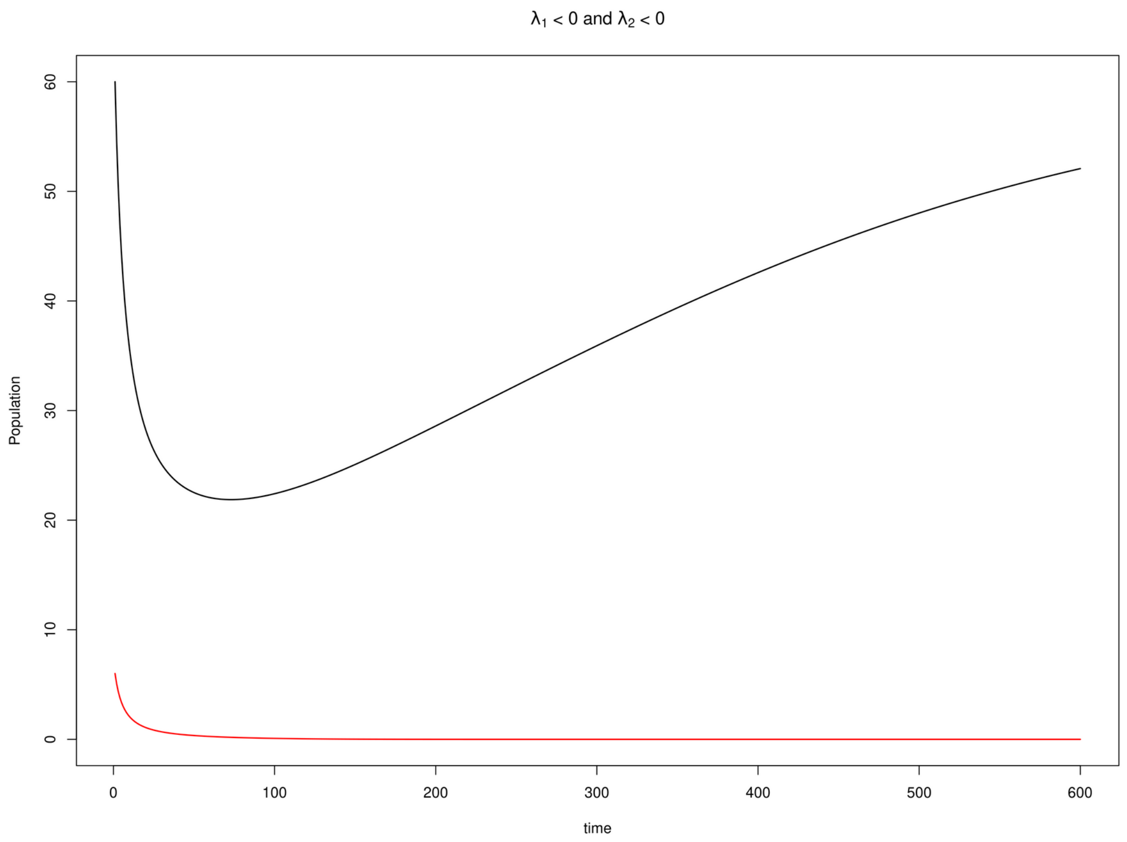

Supplementary Figure S2. With this parameters, the farmer population (red) is not able to exceed the hunter-gatherer population (black).

We can, in any case, consider the model under different equilibriums. In this regard, the dynamics of the system depend on the relative values of the competition coefficients and the carrying capacities. For example, in figure S3, we show two phase diagrams with the nullclines for farmers (blue) and hunter-gatherers (red). In the right hand side image, only farmers survive for any non-zero initial populations. On the left hand side, there is coexistence of both populations. The carrying capacity of the species, for this example, is set for hunter-gatherers to 5 and for farmers to 4. This is done with the intention to show how the model can adapt also to cases where, for specific circumstances, hunter-gatherers could have higher carrying capacities than farmers, but these are not the values used in our case studies, as already explained. In this case, growth rates are γ_{hg}=0.1 (left), γ_{hg}=0.01 (right) and γ'_f=0.05; mortality rates are δ_{hg}=0.02, δ_f=0.01, and the assimilation term η is set to =0.05. It can easily be seen that, while keeping the same model, changes in initial assumptions and parameter ranges can have a significant impact on the outcome.

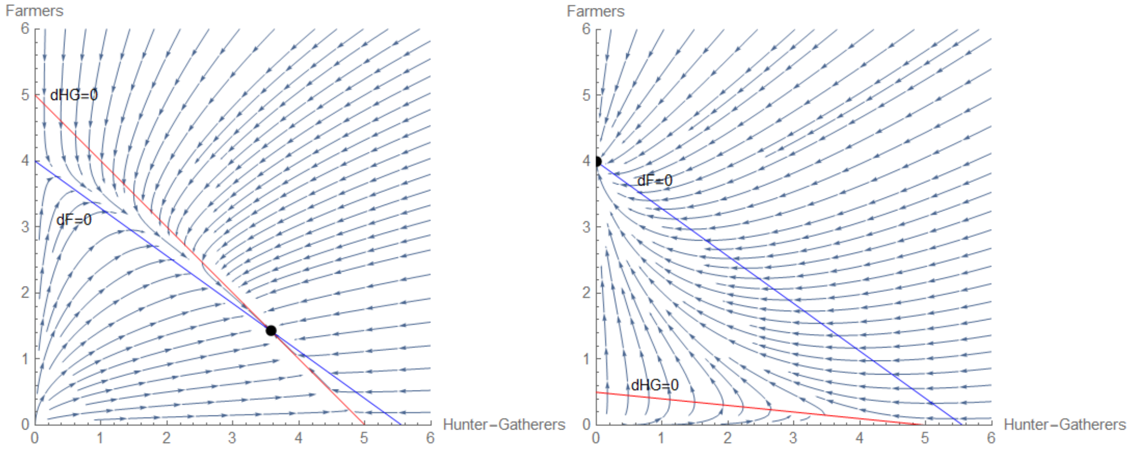

Supplementary Figure S3.

Approximate Bayesian Computation – Sequential Monte-Carlo (ABC-SMC). Due to the aforementioned difficulties to find an analytical solution. We use Approximate Bayesian Computation using a sequential Monte-Carlo algorithm. Since the method and some of its archaeological applications have already been explained elsewhere (11–14), here we only focus on how exactly we have applied the method in our specific case, and we refer to the above mentioned publications for extensive clarifications on the terminology used.

We have designed the ABC-SMC process as follows. The first thing to note is that, as our summary statistic ϵ, we use the sum of the Euclidean distances between the simulated and observed SPDs of the hunter-gatherers and the farmers. To start the ABC-SMC process we initially develop a rejection algorithm on 15000 simulations, where we select the 500 particles with the lowest ϵ to pass as the initial set of candidate values for the sequential Monte-Carlo process. This SMC process has been designed in six stages attending to the following: (1) each stage receives 500 candidate particles from the previous state and iterates until it produces 500 new accepted particles with Euclidean distances lower than ϵ; (2) ϵ is updated at the beginning of each stage and is set at the first quantile of the Euclidean distances of the 500 candidate particles and (3) in order to propose each set of parameter values from the candidate particle we use a uniform perturbation kernel $U[x-20\%, x+20\%]$ for each parameter (15).

Due to the fact that we have not set pre-specified threshold values for each stage, but compute them as the quantile of the previous candidate particles, we could increase the number of stages virtually *ad infinitum*. However, as ϵ decreases, the time needed to produce the accepted values increases. Therefore, and after simulation, we have considered six stages to be the best compromise between accuracy and efficiency
In order to avoid the problem of the ABC-SMC algorithm converging to local optima rather than global ones, we have found that it was enough to select the best 500 particles from the total generated with 100 randomised starts. The result of this is an effective recovery of the simulated (and presumably the observed) parameters, although at the cost of potentially wider posterior distributions and longer computational time.

Finally, and attending to archaeological and ethnographic literature, we have considered mainly uniform priors for our parameters, with ranges extracted from archaeological literature, often through a combination of references. The prior distributions, thus, stand as follows:

- $\gamma_{hg}\, U[0.001, 0.022]$, considering the following references: (16–20).
- $\gamma_{f}\, U[0.015, 0.035]$, considering the following references: (16, 18, 19, 21, 22).

- $\delta_{hg} U[0,0.04]$. This cannot be estimated from archaeological literature and, therefore, we have considered a slightly extended range from the growth ratio parameter, in order to stay within realistic values.
- $\delta_f U[0,0.04]$. This cannot be estimated from archaeological literature and, therefore, we have considered a slightly extended range from the growth ratio parameter, in order to stay within realistic values.
- $\eta U[0,0.3]$. Fenton et al., who implemented this parameter for ecological models, give a theoretical range of $[0,1]$, since it is essentially a proportion. We have decided to trim its upper range at 0.3 to stay within realistic levels, since a value of 1 would mean that all hunter-gatherer population would become farmers.
- $\mu U[0,0.035]$. Because of the difficulties to estimate migration rates, we have decided to set this parameter within the same range of γ_f.

Tactical simulation. We generate an SPD mimicking our process of interest, but where the parameters that created that dataset are known to us. Our intention is, first to check if we are capable of recovering the simulated population curves with our model, but also to make sure that we are able to recover the parameters of the model. By being able to recover these, we reliably trust the accuracy of the parameters on the observed dataset. Additionally, we have also used the simulation to assess (1) sample size, (2) the start and end of the interaction process, (3) total length of the process assessed and (4) efficiency and accuracy of the algorithm.
To assess sample size, we run simulations using sample sizes of 10, 25, 50, 100, 200 and 400 dates. While there is a significant improvement from 10 to 50 dates, this improvement is much less noticeable from 50 to 100 and virtually nonexistent for any dates over 100.

Regarding the start, end and length of the process, we use total interaction periods of 600, 800 and 1000 years as well as potential starting points of 100, 200, 300 and 400 years before the earliest for farmers. These have an impact on the parameters obtained. In terms of the total length, we are constrained by the shape of our observed SPDs, whereas for the starting point, we decided to set the starting of the model 100 years before the earliest farming date for all the areas. Choosing a common criterion for all of the regions has the advantage that it makes comparisons across the different regions easier. Finally, in order to recover the parameters, we have tried the option of either increasing the number of stages or randomising 100 different iterations of the full algorithm in order to avoid local optima. Despite its high computational cost, we have used the latter, since it has proven to be the most successful in recovering the pre-specified parameters.

References

1. B. Martí Oliver, J. E. Aura Tortosa, J. Juan Cabanilles, O. García Puchol, J. Fernández López de Pablo, "El Mesolítico Geométrico de tipo 'Cocina' en el País Valenciano" in *El Mesolítico Geométrico En La Península Ibérica*, P. Utrilla Miranda, L. Montes Ramírez, Eds. (Universidad de Zaragoza, 2009), pp. 205–258.
2. J. Guilaine, "The Neolithic transition in Europe: Some comments on gaps, contacts, arrythmic model, genetics" in *Unconformist Archaeology, Papers in Honour of Paolo Biagi*, British Archaeological Reports, International Series., E. Starnini, Ed. (Archaeopress, 2013).
3. T. Perrin, C. Manen, Potential interactions between Mesolithic hunter-gatherers and Neolithic farmers in the Western Mediterranean: The geochronological data revisited. *PLOS ONE* **16**, e0246964 (2021).
4. S. Shennan, *et al.*, Regional population collapse followed initial agriculture booms in mid-Holocene Europe. *Nat. Commun.* **4**, 2486 (2013).
5. P. J. Reimer, *et al.*, The IntCal20 Northern Hemisphere Radiocarbon Age Calibration Curve (0–55 cal kBP). *Radiocarbon* **62**, 725–757 (2020).

6. E. R. Crema, A. Bevan, Inference from large sets of radiocarbon dates: Software and methods. *Radiocarbon* **63**, 23–39 (2021).
7. A. J. Lotka, *Elements of Physical Biology* (Williams & Wilkins Co., 1925).
8. V. Volterra, Fluctuations in the Abundance of a Species Considered Mathematically. *Nature* **118**, 558–560 (1926).
9. A. Fenton, M. Spencer, D. J. S. Montagnes, Parameterising variable assimilation efficiency in predator–prey models. *Oikos* **119**, 1000–1010 (2010).
10. J. Hofbauer, K. Sigmund, *Evolutionary Games and Population Dynamics* (Cambridge University Press, 1998).
11. E. R. Crema, K. Edinborough, T. Kerig, S. J. Shennan, An Approximate Bayesian Computation approach for inferring patterns of cultural evolutionary change. *J. Archaeol. Sci.* **50**, 160–170 (2014).
12. K. Edinborough, E. R. Crema, T. Kerig, S. Shennan, “An ABC of lithic arrowheads: A case study from southeastern France” in *Neolithic Diversities: Perspectives from a Conference in Lund, Sweden*, 8., K. Brink, S. Hydén, K. Jennbert, L. Larsson, D. Olausson, Eds. (Acta Archaeologica Ludensia, 2015), pp. 213–224.
13. S. Carrignon, T. Brughmans, I. Romanowska, Tableware trade in the Roman East: Exploring cultural and economic transmission with agent-based modelling and approximate Bayesian computation. *PLOS ONE* **15**, e0240414 (2020).
14. A. Cortell-Nicolau, O. García-Puchol, M. Barrera-Cruz, D. García-Rivero, The spread of agriculture in Iberia through Approximate Bayesian Computation and Neolithic projectile tools. *PLOS ONE* **16**, e0261813 (2021).
15. T. Toni, D. Welch, N. Strelkowa, A. Ipsen, M. P. H. Stumpf, Approximate Bayesian computation scheme for parameter inference and model selection in dynamical systems. *J. R. Soc. Interface* **6**, 187–202 (2009).
16. J. Fort, J. Pérez-Losada, J. J. Suñol, L. Escoda, J. M. Massaneda, Integro-difference equations for interacting species and the Neolithic transition. *New J. Phys.* **10** (2008).
17. N. Isern, J. Fort, A. F. Carvalho, J. F. Gibaja, J. J. Ibañez, The Neolithic Transition in the Iberian Peninsula: Data Analysis and Modeling. *J. Archaeol. Method Theory* **21**, 447–460 (2014).
18. M. Currat, L. Excoffier, The effect of the Neolithic expansion on European molecular diversity. *Proc. Biol. Sci.* **272**, 679–688 (2005).
19. A. J. Ammerman, L. L. Cavalli-Sforza, *The Neolithic Transition and the Genetics of Populations in Europe* (Princeton University Press, 1984).
20. M. Jackes, M. Roksandic, C. Meiklejohn, “Demography of the Djerdap Mesolithic-Neolithic transition” in *The Iron Gates in Prehistory: New Perspectives*, C. Bonsall, V. Boroneant, I. Radovanovic, Eds. (Archaeopress, 2008), pp. 77–88.
21. J. Fort, V. Méndez, Time-Delayed Theory of the Neolithic Transition in Europe. *Phys. Rev. Lett.* **82**, 867–870 (1999).
22. N. Isern, J. Zilhão, J. Fort, A. J. Ammerman, Modeling the role of voyaging in the coastal spread of the Early Neolithic in the West Mediterranean. *Proc. Natl. Acad. Sci. U. S. A.* **114**, 897–902 (2017).

SUPPLEMENTARY MATERIAL 2 (S2)

Results of Tactical Simulation

In order to assess the validity of our model, we initially deployed a tactical simulation (1, 2) to check whether we could recover (1) the shape of the observed SPDs and (2) the LV parameters generating the underlying populations (see Methods and Supplementary Information for details). Fig. 1 shows how our inferential tool can successfully recover the 'true' model parameters. It is worth noting that despite the fact that LV models are designed to account for the interaction between two existing populations, they usually refer to populations in equilibrium (where both of them survive). However, due to the modification included in the theoretical model (see methods and S1), we are still able to recover the original demographic process even for long periods without a noticeable presence of the farming population. This is particularly relevant for some of our case studies, where the limited sample sizes can potentially give the impression of the absence of initial migrant communities that are undetectable archaeologically because of their small numbers. Alternatively, this implies considering an earlier arrival of farming pioneers (3, 4).

We explore the model with different sample sizes. Except for the cases with an extremely low number of radiocarbon dates (e.g. 10 or 25 radiocarbon dates), the model is able to recover the true parameters, with little improvement on the precision of its highest posterior density interval (HDPI) from 50 to 100 radiocarbon dates, and almost none at all above that number. The posterior distributions of our parameters in the simulation recapture the original values both at a 95% and at an 80% HDPI. The recovery of the parameters δ_{hg}, γ_{hg} and δ_f seems quite robust, despite the long tails of the two latter ones, and the recovery of γ'_f looks also acceptable, albeit a bit more platykurtic, probably due to the hierarchical nature of the parameter, which combines migration and growth rate. On the other hand, the posterior distribution of η, although successfully recovering the original value, is undoubtedly wider and can pose problems for further interpretation.

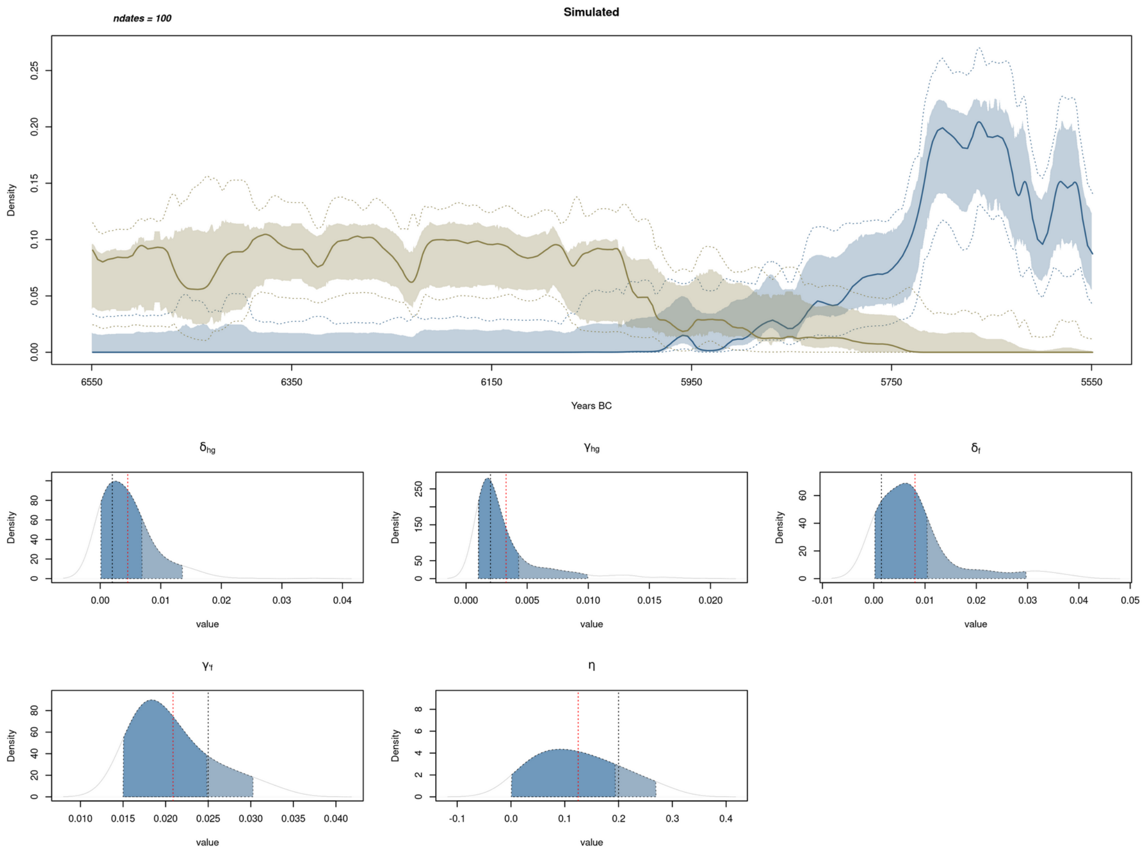

Supplementary figure S4. Posterior predictive check and posterior distributions of the parameters, indicating the target mean (black dashed-line) and the mean of the distribution (red dashed-line), for the simulated dataset. On the fitted SPD, lines indicate the target simulated SPD and lighter colour represent the 95% HDPI. On the parameters, lighter blue represents the 95% HDPI and darker blue represents the 80% HDPI.

References:

1. C. Orton, "The tactical use of models in archaeology - the SHERD project" in *The Explanation of Culture Change*, C. Renfrew, Ed. (Duckworth, 1973), pp. 137–139.
2. M. W. Lake, Trends in Archaeological Simulation. *J. Archaeol. Method Theory* **21**, 258–287 (2014).
3. F. J. J. Jover Maestre, G. G. García Atiénzar, Sobre la neolitización de los grupos mesolíticos en el este de la Península Ibérica: la exclusión como posibilidad. *Pyrenae* **45**, 55–88 (2014).
4. C. Manen, *et al.*, Le sommet de l'iceberg ? Colonisation pionnière et néolithisation de la France méditerranéenne. *Bull. Société Préhistorique Fr.* **116**, 317–361 (2019).

SUPPLEMENTARY MATERIAL 3 (S3)

Additional plots for parameters exploration

Experiment 1: Explore γ_{hg} and γ'_f while δ_{hg} and δ_f remain constant at 0.01.

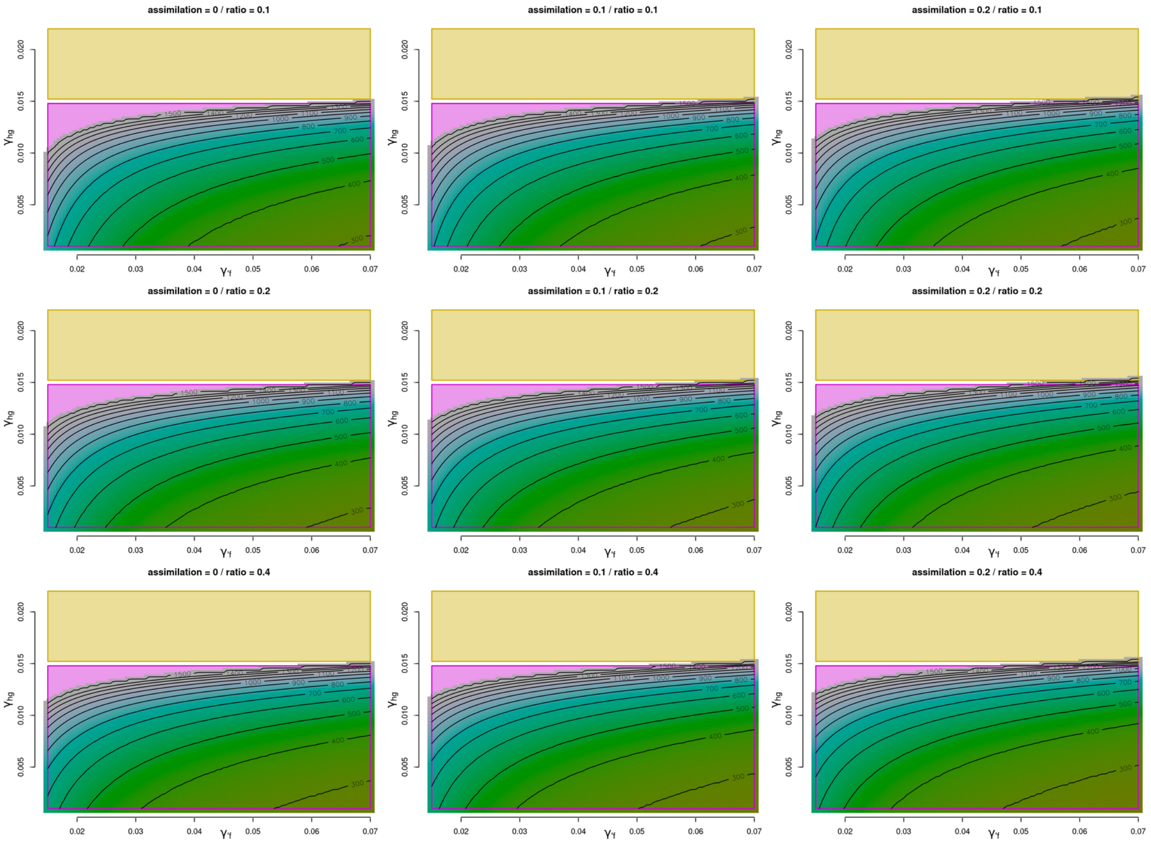

Supplementary figure S5. Time taken for the farmers to reach their carrying capacity under the full range of the growth parameters. Interspecific mortality parameter is set to 0.01 for both populations, assimilation parameter and initial population ratio follow the specifications of table 1 in the main manuscript. Magenta indicates the parametric area where the farmers will be the only surviving population and yellow area indicates coexistence.

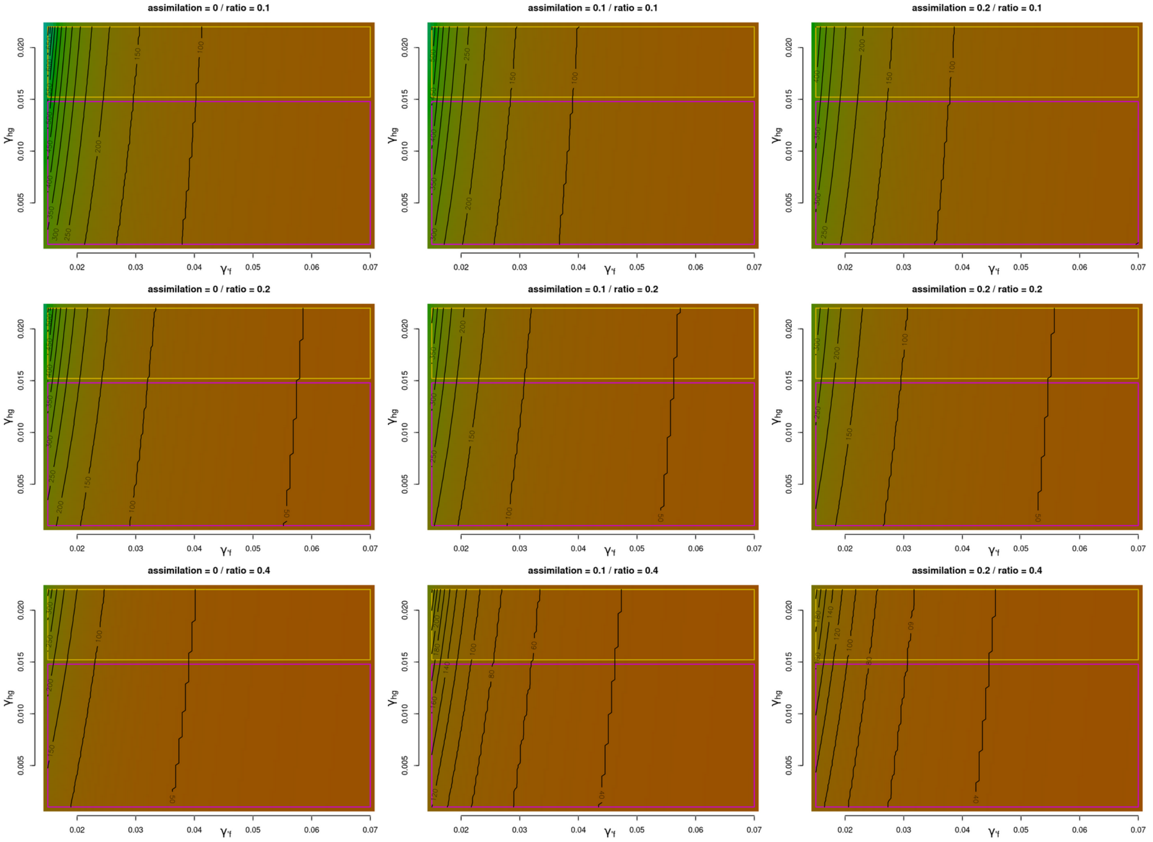

Supplementary figure S6. Time taken for the farmers to exceed the hunter-gatherer population under the full range of the growth parameters. Interspecific mortality parameter is set to 0.01 for both populations, assimilation parameter and initial population ratio follow the specifications of table 1 in the main manuscript. Magenta indicates the parametric area where the farmers will be the only surviving population and yellow area indicates coexistence.

Experiment 2: Explore δ_{hg} and δ_f while γ_{hg} and γ'_f remain constant at 0.015 and 0.02 respectively.

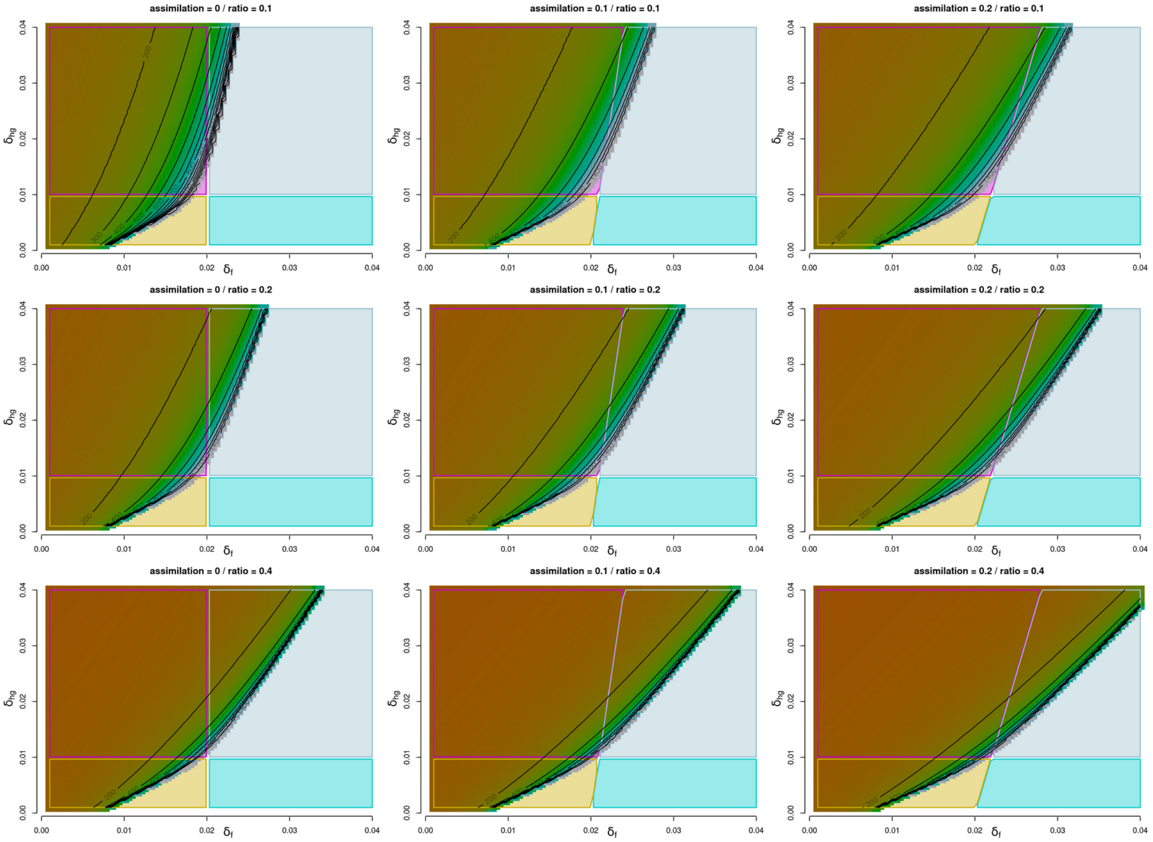

Supplementary Figure S7. Time taken for the farmers to surpass the hunter-gatherer population under the full range of the interspecific mortality parameters. Growth rate parameter is set to 0.015 for the hunter-gatherers and 0.02 for the farmers. Assimilation parameter and initial population ratio follow the specifications of table 1 in the main manuscript. Magenta indicates the parametric area where the farmers will be the only surviving population, yellow area indicates coexistence, in the blue area only the hunter-gatherers will survive and in the grey area only one population survives, and which one does depends on the initial conditions.

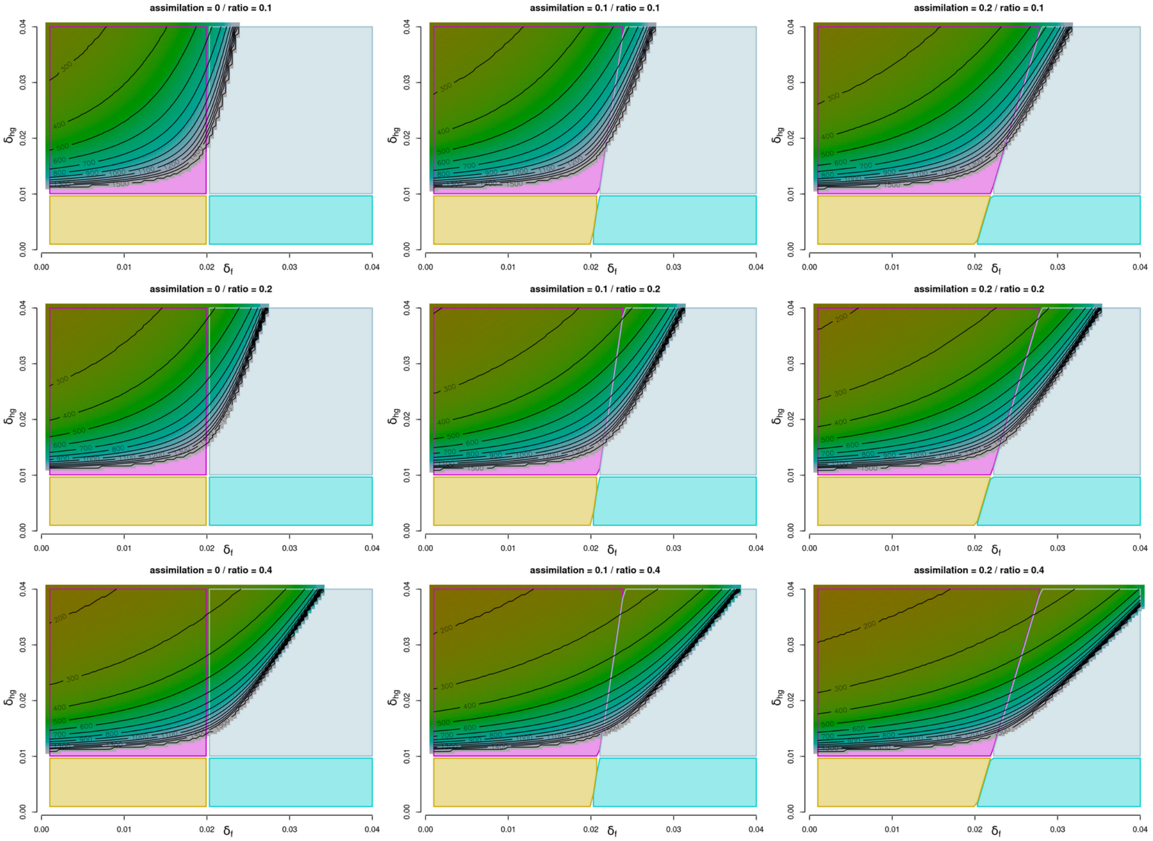

Supplementary Figure S8. Time taken for the hunter-gatherers to disappear under the full range of the interspecific mortality parameters. Growth rate parameter is set to 0.015 for the hunter-gatherers and 0.02 for the farmers. Assimilation parameter and initial population ratio follow the specifications of table 1 in the main manuscript. Magenta indicates the parametric area where the farmers will be the only surviving population, yellow area indicates coexistence, in the blue area only the hunter-gatherers will survive and in the grey area only one population survives, and which one does depends on the initial conditions.
